# Profiling Synaptic Proteins Identifies Regulators of Insulin Secretion and Lifespan

**DOI:** 10.1371/journal.pgen.1000283

**Published:** 2008-11-28

**Authors:** QueeLim Ch'ng, Derek Sieburth, Joshua M. Kaplan

**Affiliations:** Department of Molecular Biology, Massachusetts General Hospital, Boston, Massachusetts, United States of America; Stanford University Medical Center, United States of America

## Abstract

Cells are organized into distinct compartments to perform specific tasks with spatial precision. In neurons, presynaptic specializations are biochemically complex subcellular structures dedicated to neurotransmitter secretion. Activity-dependent changes in the abundance of presynaptic proteins are thought to endow synapses with different functional states; however, relatively little is known about the rules that govern changes in the composition of presynaptic terminals. We describe a genetic strategy to systematically analyze protein localization at *Caenorhabditis elegans* presynaptic specializations. Nine presynaptic proteins were GFP-tagged, allowing visualization of multiple presynaptic structures. Changes in the distribution and abundance of these proteins were quantified in 25 mutants that alter different aspects of neurotransmission. Global analysis of these data identified novel relationships between particular presynaptic components and provides a new method to compare gene functions by identifying shared protein localization phenotypes. Using this strategy, we identified several genes that regulate secretion of insulin-like growth factors (IGFs) and influence lifespan in a manner dependent on insulin/IGF signaling.

## Author Summary

Cells are divided into multiple subcellular compartments that perform diverse functions. In neurons, synapses mediate transmission of information between cells and they comprise hundreds of proteins dedicated for this purpose. Changes in the protein composition of synapses are thought to produce changes in synaptic transmission, such as those that occur during development, learning, and memory. Here, we describe a systematic genetic strategy for analyzing the protein composition of synapses. Using this strategy, we identified sets of genes that alter synapses in similar ways, and identified novel regulatory relationships between particular synaptic proteins. One set of genes regulated secretion of insulin-like hormones from neurons and had corresponding effects on lifespan, which is controlled by insulin signaling. These results illustrate how changes in synaptic composition can be utilized as a probe to explain changes in physiology. Our approach can be expanded to include a larger set of synaptic proteins or to analyze other subcellular compartments.

## Introduction

Differentiation and organization of eukaryotic cells require regulated localization of specific proteins into subcellular compartments where they perform discrete functions. Global analysis of protein localization in yeast revealed that >40% of proteins localize to specific subcellular compartments [1]. This large repertoire of localized proteins raises several questions. What genes or pathways orchestrate the subcellular abundance and distribution of these proteins? Is the protein composition of subcellular compartments static or plastic? What rules govern the composition of these structures? How do changes in protein localization alter the function of these structures, and ultimately organismal health?

The large variety of proteins in subcellular compartments implies substantial genetic and biochemical complexity. Therefore, addressing these questions will require comprehensive and systematic approaches beyond the study of single genes or proteins. Such approaches have been successful in identifying groups of functionally related genes based on similarities in their expression patterns, or similarities in the phenotypic consequences of disrupting gene function [2]–[4]. Given recent advances in high-content imaging screens [1], it is now possible to do analogous studies linking gene function to changes in the global patterns of protein localization.

In neurons, presynaptic specializations are estimated to contain approximately one thousand proteins that are configured into discrete compartments; these compartments contain different organelles and perform different cellular functions in neurotransmitter release [5]. Synaptic vesicles (SVs) contain neurotransmitters that are released upon SV fusion with the plasma membrane. The active zones are structures containing many scaffold proteins and calcium channels and are sites of SV fusion. Periactive zones are F-actin-rich areas where SV recycling occurs through endocytosis. Dense core vesicles (DCVs) are vesicles that release neuropeptides and peptide hormones [6], including insulin/IGF ligands implicated in metabolic diseases and the aging process [7],[8]. DCVs are a population of vesicles distinct from SVs that undergo differential regulated release at different locations in neurons [6]. DCVs have mostly been studied in cultured secretory cells; thus, genetic factors that regulate DCV secretion in vivo from neurons are poorly understood, despite their importance in health and disease.

Synapses are able to operate over a broad range of functional states, which endow circuits with the capacity to store and process information. Relatively little is known about how the protein composition of synapses is altered across these functional states, nor how these changes contribute to differences in synaptic transmission. Is the abundance of proteins associated with the same subsynaptic structure (e.g. SVs) always correlated across physiological states? To what extent do the binary interactions between presynaptic proteins govern changes in presynaptic composition? Can changes in protein localization profiles be linked to changes in behavior and physiology of the whole animal?

Here, we describe a genetic analysis of presynaptic structure in *C. elegans* by measuring in vivo changes in the abundance and distribution of a panel of fluorescently tagged presynaptic proteins. These proteins label distinct subsynaptic compartments and are involved in diverse aspects of neurotransmitter release (Table 1). Using these markers, we determined how synapse structure was altered in twenty-five mutants that alter various aspects of synaptic transmission. By comparing changes in protein localization caused by different mutations, we describe changes in protein composition of presynaptic terminals across a range of physiological states. In this manner, we identify several genes that regulate secretion of insulin/IGFs from neurons, and we show that these genes regulate lifespan, a physiological function of IGF signaling.

**Table 1:** List of markers and compartments labeled.

Marker	Ortholog	Localization	Reference(s)
GFP::SNB-1	Synaptobrevin	SVs, plasma membrane	[11],[80]
GFP::SYD-2	α-Liprin	Active zone	[11],[30],[31],[81]
SNN-1::Venus	Synapsin	Perisynaptic regions	[11],[44],[82]
UNC-10::GFP	RIM1α	Active zone	[11],[35]
Venus::RAB-3	Rab3	SVs	[11],[42],[53]
Gelsolin::Venus	Gelsolin	Barbed ends of F-actin, perisynaptic regions	[11],[44],[83]
APT-4::GFP	AP2 α-adaptin	Endocytic sites	[11],[84]
INS-22:Venus	Insulin/IGF	DCVs	[11],[52]
ITSN-1::GFP	Intersectin/DAP160	Endocytic sites	[46]–[50]

Location of the fluorescent tag is indicated by the order of the tag and the protein. N-terminal tagged proteins are preceded by GFP or Venus, C-terminal tagged proteins are followed by GFP or Venus.

## Results

### Quantitative Imaging of Nine Presynaptic Proteins

**Figure 1:**
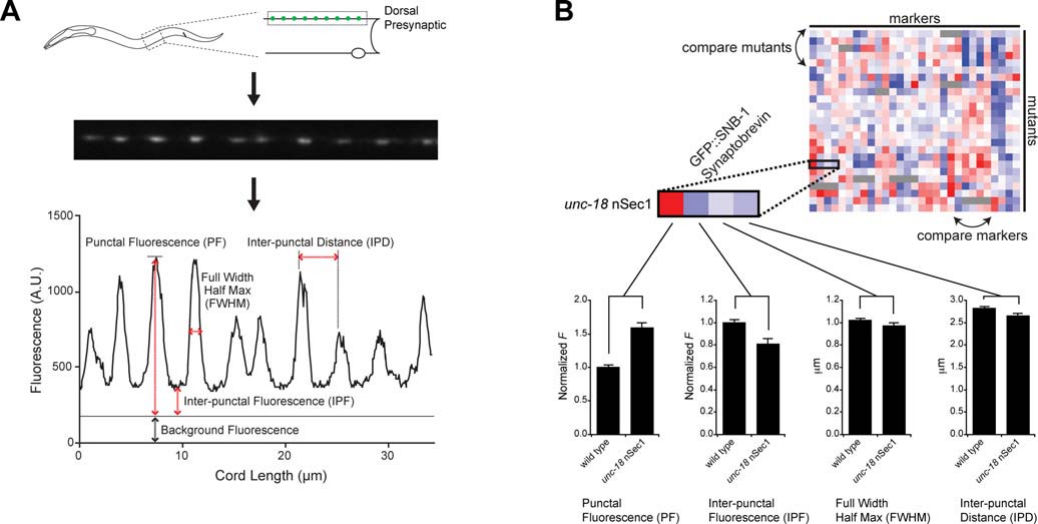
In vivo imaging of synaptic proteins. (A) Top: Imaging presynaptic specializations in dorsal axons at the NMJ. Middle: Fluorescence image of SNB-1 synaptobrevin in wild type animals. Each punctum represents a cluster of SV at a presynaptic terminal. Bottom: A trace representing pixel fluorescence values along the axon. Parameters analyzed in this study are indicated. (B) Representation of changes observed in the four parameters for each synaptic marker in each mutant background tested. Changes in each parameter are expressed as a continuous score reflecting the magnitude and significance of the change between mutant and the corresponding wild type control samples based on the Student's T-statistic. Positive scores (red shading) and negative scores (blue shading) indicate an increase or decrease respectively in a given parameter in the mutant compared to wild type. The magnitude of the score is indicated by the intensity of the shading. How *unc-18* nSec1 mutants affected SNB-1 synaptobrevin is used as an example. Error bars are ±SEM.

To explore the networks of interactions between presynaptic proteins in *C. elegans*, we generated a panel of nine markers that label different presynaptic compartments, including SVs, DCVs, active zones, endocytic vesicles/sites and actin cytoskeleton (Table 1). We constructed stable chromosomally integrated transgenes consisting of these markers tagged with GFP or Venus/YFP [9] and expressed in the DA class of cholinergic motor neurons that form presynaptic terminals at body wall neuromuscular junctions (NMJs) (Figure 1A) [10] . Because axons of these motor neurons form en-passant synapses with body wall muscles, synaptic proteins adopt a punctate pattern of localization along the length of the axon (Figure 1A). Prior studies have shown that the fluorescent puncta formed by these tagged proteins correspond to presynaptic specializations [11] (Table 1). To quantify the abundance and distribution of these markers in axons, we averaged fluorescence from ∼300–600 synapses (from ∼30 animals). Using custom software, we determined four parameters: the punctal fluorescence, which measures abundance at presynaptic specializations; inter-punctal fluorescence, which measures axonal abundance between synapses; full width at half maximum (FWHM), which measures punctal width; and inter-punctal distance, which measures the distance between synapses along the axon (Figure 1A) [12],[13]. For some markers, a subset of these parameters was excluded from our analysis (see Materials and Methods). Puncta widths were excluded for SYD-2 α-Liprin, SNN-1 Synapsin, and UNC-10 RIM1 because these values were close to the diffraction limit, and thus changes in widths could not be accurately measured. Similarly, the interpunctal fluorescence values observed in SYD-2 α-Liprin, UNC-10 RIM1α, and APT-4 α2-adaptin were not significantly different from background fluorescence, and consequently were excluded from our analysis.

**Figure 2:**
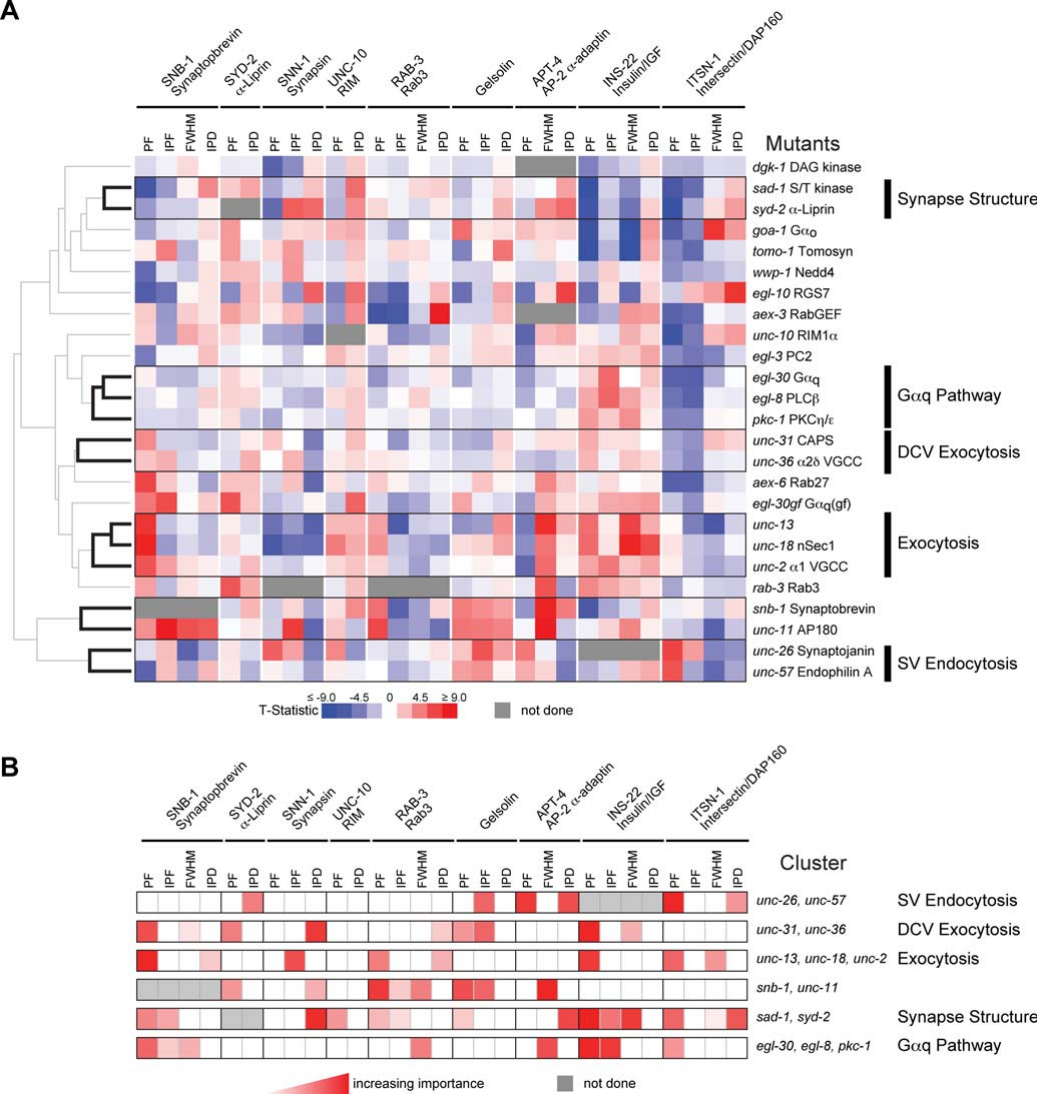
Clustering analysis of synaptic proteins and synaptic transmission mutants. (A) Phenotypic clustering of mutants. Each row represents the protein localization profile of a single mutant corresponding to the indicated gene. Increases and decreases in the parameters measured (see text and Figure 1B) are represented in red and blue respectively, and the magnitudes of the changes are indicated by the intensity. Black branches in the dendrogram and boxed areas indicate robust and statistically significant clusters (*p*<0.05 with Bonferroni Correction). The function of the genes in a cluster is indicated to the right. PF = punctal fluorescence, IPF = Inter-punctal fluorescence, FWHM = Full Width at Half Maximal, IPD = Inter-punctal Distance. IPF was not analyzed in some protein markers (e.g. SYD-2 α-Liprin) because it was not significantly above background. FWHM was not analyzed for markers where the puncta were diffraction limited (e.g. UNC-10 RIM1α). (B) Shared phenotypes for each cluster. Each row represents the analysis of a single cluster. The contribution of each parameter to the grouping of each cluster, as calculated by the amount their exclusion reduced the strength of correlations between phenotypic profiles within each cluster is indicated in red. (See Materials and Methods and Supporting Information in Text S1.) Darker shading indicates increasing importance of the parameter for the grouping of the cluster. The genes in each cluster and their function are indicated to the right.

To determine how presynaptic composition is altered across a range of conditions that alter synaptic function, these presynaptic markers were crossed into each of twenty-five neurotransmission mutants, excluding cases where the marker and mutation corresponded to the same gene or when the marker and mutation were too closely linked to isolate recombinants. In this manner, we produced a total of 218 marker/mutant combinations, which we analyzed for phenotypes in the localization of synaptic markers to obtain the protein localization profiles of these twenty-five mutants (Figure 2A). We analyzed fluorescence changes for each presynaptic marker compared to wild-type controls. For a given synaptic marker, differences between the mutant and wild type samples for each parameter were quantified using the T-statistic (Figure 1B, Table S3). The pattern of changes for all nine synaptic markers caused by a mutant constitutes a “protein localization profile,” which provides a description of how synapse structure is altered by mutations in a particular gene.

The mutations selected for this analysis affect diverse aspects of synaptic transmission, including G-protein signaling pathways and components of exocytic or endocytic machinery involved in the SV cycle. Some of these mutations are well characterized, based on previous behavioral, electrophysiological or ultrastructural studies. These well-characterized mutations served as positive controls to validate our approach, and provide canonical protein localization profiles for comparison to less-characterized mutations. The majority of the mutations we analyzed decrease neurotransmission, but we also selected four mutations that increase neurotransmission (*dgk-1* DAG kinase, *goa-1* Gα_o_, *tomo-1* Tomosyn, and a constitutively active form of *egl-30* Gα_q_) [14]–[22]. Thus, our synaptic protein localization profiles allow us to describe changes in synaptic protein localization that occur following bidirectional changes in neurotransmission.

### Analyzing Synaptic Protein Localization Profiles

Synaptic protein localization profiles capture functional relationships between different genes and different presynaptic proteins. Several kinds of regulatory relationships are observed in this dataset. First, the effect of a single mutation on an individual marker can indicate a functional relationship between a gene and protein. Second, at the level of the whole dataset, similarities between mutant protein localization profiles or marker protein profiles might reveal related functions or interactions. Third, trends and potential outliers identified in the dataset may represent specific pathway(s) required to coordinate particular aspects of synaptic function. Fourth, this dataset could be used as a basis for classifying uncharacterized genes. We provide several examples to illustrate these analytical techniques in the following sections.

### Hierarchical Clustering of Protein Localization Profiles

We used hierarchical clustering to identify groups of related genes based on similarities among their protein localization profiles. Six gene clusters were detected robustly across multiple clustering strategies and consisted of profiles that were significantly and positively correlated (see below) (Figure 2A, Table S1). To gain insight into the function of the genes in each cluster, we determined which shared phenotypes contributed most significantly to the positive correlation between the profiles within each cluster (Figure 2B) (see Materials and Methods and Supporting Information in Text S1). We found that proteins within different clusters had distinct shared phenotypes, confirming that each cluster affected distinct cellular processes.

This analysis identified three clusters consisting of genes previously reported to have related functions in neurotransmission, thereby validating this approach (Figure 2A). One cluster contained two genes involved in SV endocytosis, *unc-26* synaptojanin and *unc-57* endophilin A (Figure 2A) [23],[24]. During endocytosis, UNC-26 synaptojanin is recruited to endocytic vesicles by UNC-57 endophilin A [24],[25]. Prior ultrastructural studies have shown that endocytic intermediates (e.g. clathrin coated pits and vesicles) accumulate in *unc-57* endophilin A and *unc-26* synaptojanin mutant synapses [23],[24]. To confirm that our profiling strategy can detect this aspect of the *unc-26* synaptojanin and *unc-57* endophilin A mutant phenotypes, we analyzed two proteins that label endocytic vesicles: the α2 subunit of the AP2 clathrin adaptin (APT-4) and intersectin/DAP160 (ITSN-1) (Table 1). We observed increased punctal fluorescence of APT-4 α2 adaptin and ITSN-1 intersectin in *unc-26* synaptojanin and *unc-57* endophilin A mutant synapses (Table S2), consistent with the accumulation of endocytic intermediates in these mutants [23],[24]. Moreover, these phenotypes contributed most to the clustering of these two genes (Figure 2B). Thus, identifying shared phenotypes can verify the related functions of genes in a cluster.

A second cluster was comprised of three genes required for exocytosis, *unc-13*, *unc-18* nSec1, *unc-2* α1 voltage gated calcium channel (VGCC) subunit [26]–[28]. These genes clustered together because of increased punctal fluorescence of SV (SNB-1 synaptobrevin and RAB-3) and DCV (INS-22 insulin/IGF) proteins and because they did not strongly affect an endocytic protein (ITSN-1 intersectin/DAP160) (Figure 2A–B). The increased SNB-1 and RAB-3 punctal fluorescence observed suggests that SVs accumulate in these mutants, consistent with prior ultrastructural studies [26],[28].

A third cluster comprised two genes involved in synapse formation, *syd-2* α-Liprin and *sad-1* kinase [29]–[31]. These genes clustered because of significant reductions in synapse numbers (increased inter-punctal distance of several markers) and defects in presynaptic morphology (decreased punctal fluorescence of several markers) in these mutants compared to wild type (Figure 2A–B). The defects in presynaptic morphology that we observed in the DA motor neurons of the *syd-2* α-Liprin and *sad-1* kinase mutants are similar to those previously described in other classes of neurons [29]–[31]. Thus, presynaptic protein localization profiles can be used to organize genes into groups with shared phenotypes, which may indicate related gene functions.

### Comparing Mutants Based on Protein Localization Changes

**Figure 3:**
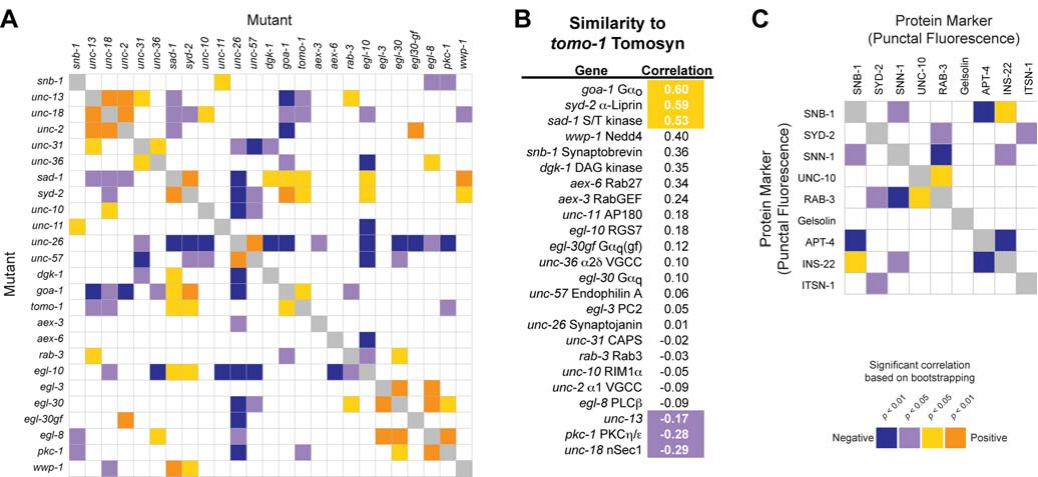
Correlation analysis among mutants and markers. (A) Correlations between the phenotypic profiles of mutants analyzed. Pairwise Pearson's Correlation Coefficients were calculated between all mutations tested, with significant positive or negative correlations indicated by shaded boxes according to the legend in (C). (B) Genes ranked by similarity to an example query gene, *tomo-1* tomosyn, based on their correlation values to *tomo-1* tomosyn; higher positive correlation indicates greater similarity. Significant positive and negative correlations based on bootstrapping (Material and Methods) are shaded according to the legend in (C). (C) Correlations between the marker profiles of presynaptic markers. Significant correlations are shaded as indicated in the legend. For comparing markers, we analyzed the punctal fluorescence as a measure of presynaptic abundance in each mutant background and compared all pairwise combinations.

Hierarchical clustering utilizes positive correlations to generate a single representation of relationships among genes. For this reason, certain kinds of information are not represented in hierarchical clustering strategies. First, significant similarities beyond those within the gene clusters are not illustrated. Second, mutations in two genes may have opposite phenotypic effects on synapse structure, which would lead to anti-correlated phenotypes. To address these issues, we made pairwise comparisons of all twenty-five mutant profiles using the Pearson's Correlation to measure similarity (Figures 3A). The significance of the correlation coefficients was determined using a bootstrapping approach (see Materials and Methods).

In this manner, we identified similar or opposite phenotypes among the mutants tested (Figure 3A). As expected, positive correlations were observed between protein localization profiles that mirror the results from the hierarchical clustering. For example, a positive correlation was observed between *unc-13*, *unc-18* nSec1, and *unc-2* (Figure 3A). A positive correlation was also observed between two genes that function to inhibit neurotransmitter secretion: *tomo-1* tomosyn and *goa-1* Gα_o_ (Figure 3A–B) [14]–[17], [19]–[21]. Interestingly, the protein localization profiles of the exocytosis genes (*unc-13* and *unc-18* nSec1) were anti-correlated with those of genes that inhibit exocytosis (*tomo-1* tomosyn and *goa-1* Gα_o_) (Figure 3A–B). Thus, the markers used here provide bidirectional information about genes that affect neurotransmitter release, and illustrate how anti-correlations can provide useful information about gene functions.

### Correlated Changes in Presynaptic Markers

To identify relationships between pairs of protein markers, we conducted systematic pairwise comparisons of the punctal fluorescence of each protein, using the Pearson's Correlation to measure similarity (Figure 3C). Most of the marker profiles determined in this manner were not correlated, suggesting that the corresponding presynaptic proteins are regulated independently. For several presynaptic proteins, we observed significant positive or negative correlations. These marker correlations suggest regulatory relationships among these proteins. For example, one might expect positive correlations to be observed for proteins involved in the same process (e.g. SV exo- or endocytosis), those that associate with the same presynaptic organelle (e.g. SVs), or those that bind to each other. Several examples of this analysis are described below.

### Comparing Active Zone Proteins

**Figure 4:**
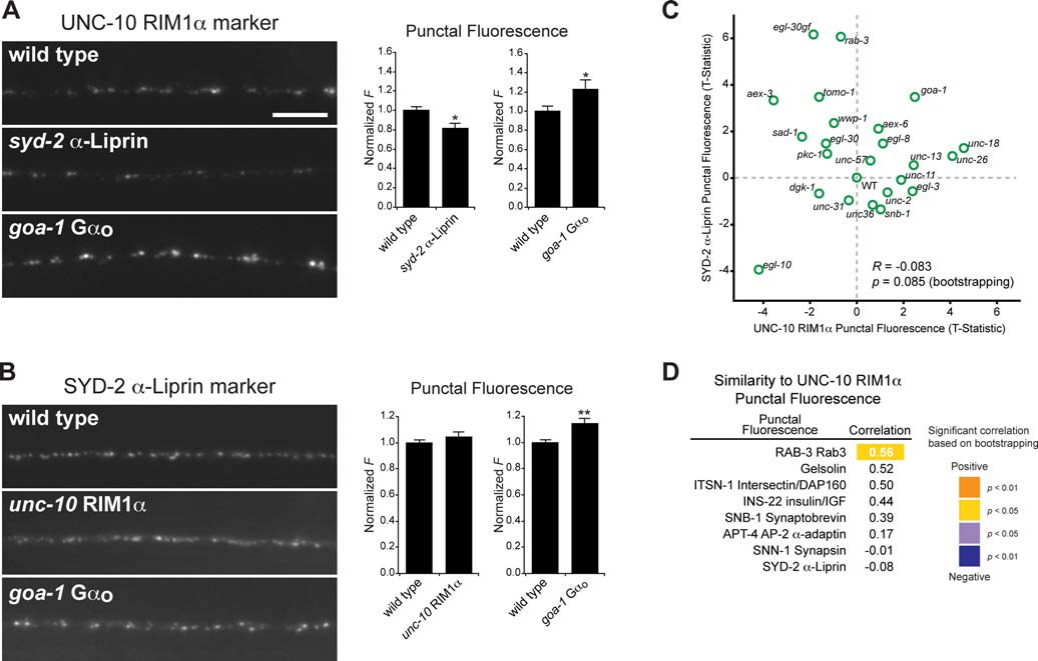
Analysis of active zones. (A) Images of UNC-10 RIM1α in axons of wild type and the indicated mutant animals. Scale bar, 5 µm. Quantification of UNC-10 RIM1α punctal fluorescence is shown to the right. (B) Images of SYD-2 α-Liprin in axons of wild type and the indicated mutant animals. Quantification of SYD-2 α-Liprin punctal fluorescence is shown to the right. In (A–B), * indicates *p*<0.05, ** indicates *p*<0.01, (Student's T-Test compared to wild type). All error bars are ±SEM. Separate charts indicate data from separate sets of experiments. (C) XY plot revealing lack of correlation between changes in the punctal fluorescence of SYD-2 α-Liprin and UNC-10 RIM1α. (D) List of markers ranked by similarity to UNC-10 RIM1α based on their punctal fluorescence.

The active zone is a complex matrix of proteins that are enriched at sites of SV fusion. Many biochemical interactions have been observed among active zone proteins, and these interactions are thought to regulate recruitment of these proteins to synapses, e.g. during synapse formation or synaptic plasticity. We analyzed two active zone proteins that are binding partners, UNC-10 RIM1α and SYD-2 α-Liprin [32]. One possible function for their biochemical interaction is the assembly of active zone components. Consistent with this idea, we found that UNC-10 RIM1α punctal fluorescence was significantly reduced in *syd-2* α-Liprin mutants (Figure 4A); however, SYD-2 α-Liprin punctal fluorescence was not reduced in *unc-10* RIM1α mutants (Figure 4B), both in agreement with prior work [30],[33]. These results suggest that SYD-2 α-Liprin is involved in recruiting UNC-10 RIM1α to synapses, but does not require UNC-10 RIM1α for normal presynaptic localization. Furthermore, SYD-2 α-Liprin and UNC-10 RIM1α punctal fluorescence both increased in *goa-1* Gα_o_ mutants, suggesting that they can be coordinately regulated (Figure 4A–B). Prior studies showed that *goa-1* Gα_o_ also regulates the abundance of UNC-13 [21], another active zone protein that binds UNC-10 RIM1α. Taken together, these results suggest that *goa-1* Gα_o_ coordinately regulates the synaptic abundance of several interacting active zone proteins.

UNC-10 RIM1α is a scaffolding protein with many potential binding partners besides SYD-2 α-Liprin [34]. If SYD-2 α-Liprin was the primary determinant of UNC-10 RIM1α localization, we would expect that their abundance would be positively correlated in our dataset. Contrary to this prediction, we found no significant correlation in their fluorescence across the 25 mutants analyzed (*R* = −0.083, *p* = 0.085) (Figure 4C–D). In fact, several mutations had opposite effects on SYD-2 α-Liprin and UNC-10 RIM1α punctal fluorescence. For example, three mutants that had large increases in SYD-2 α-Liprin fluorescence, *egl-30(gf)* constitutively active Gα_q_, *aex-3* RabGEF, and *tomo-1* tomosyn, all had significantly reduced UNC-10 RIM1α punctal fluorescence (Figure 4C, Table S2). Taken together, these results indicate that the synaptic abundance of UNC-10 RIM1α and SYD-2 α-Liprin are largely regulated independently across the mutants tested. Consistent with this notion, UNC-10 RIM1α can still localize to discrete puncta in SYD-2 α-Liprin null mutants, albeit less efficiently (Figure 4A), suggesting that SYD-2 α-Liprin is not the sole determinant of UNC-10 RIM1α localization.

In worm and mouse knockouts lacking RIM1α, SV fusion is impaired but not eliminated [34],[35]. In *unc-10* RIM1α mutant worms, the reduced SV fusion rate is accompanied by decreased SV docking and priming [34]–[36]. In mammals, RIM1α binds to GTP-bound RAB-3, a Ras-related GTPase involved in SV exocytosis. The analogous proteins in *C. elegans*, UNC-10 and RAB-3, are also binding partners [37], consistent with the significant positive correlation between the punctal fluorescence changes for RAB-3 and UNC-10 RIM1α in our dataset (Figure 4D) (R = 0.56; *p* = 0.049) that indicate UNC-10 RIM1α and RAB-3 synaptic abundance are coordinately regulated.

### Comparing SV Proteins

**Figure 5:**
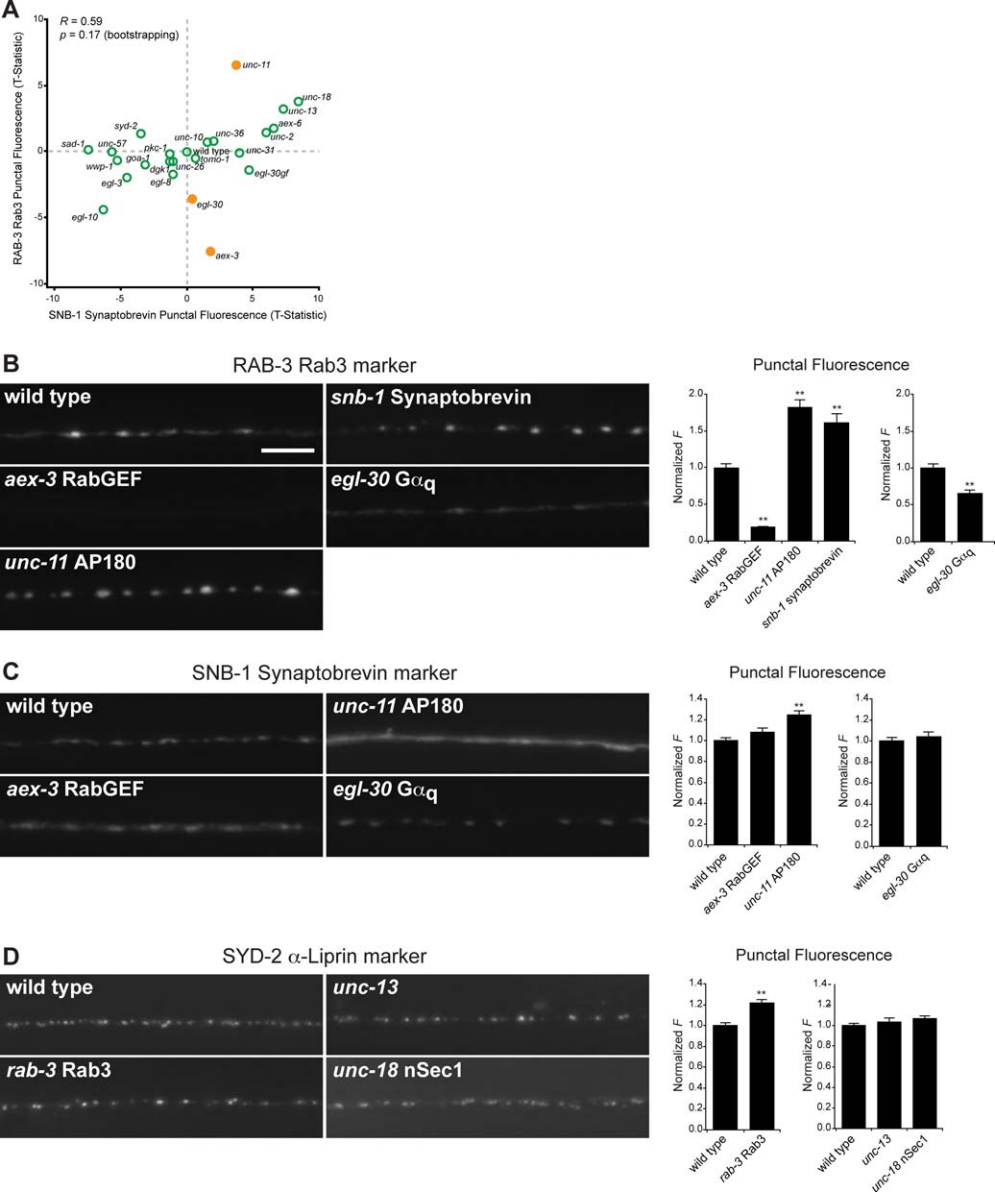
Analysis of SV and active zone markers. (A) XY plot comparing changes in punctal fluorescence for SV markers: RAB-3 vs. SNB-1 synaptobrevin across mutants tested. Solid orange circles indicate mutants that are apparent outliers described in the text. (B) Images of RAB-3 in axons of wild type and the indicated mutant animals. Quantification of RAB-3 punctal fluorescence is shown to the right. Scale bar, 5 µm. (C) Images of the SNB-1 synaptobrevin in axons of wild type and the indicated mutant animals. Quantification of SNB-1 synaptobrevin punctal fluorescence is shown to the right. (D) Images of SYD-2 α-Liprin in axons of wild type and the indicated mutant animals. Quantification of SYD-2 α-Liprin punctal fluorescence is shown to the right. In (B–D), ** indicates *p*<0.01, (Student's T-Test compared to wild type). All error bars are ±SEM. Separate charts indicate data from separate sets of experiments.

SNB-1 Synaptobrevin and RAB-3 are two proteins associated with SVs and both are required for normal levels of SV exocytosis. To determine whether the synaptic abundance of these two proteins are differentially regulated, we plotted the punctal fluorescence of RAB-3 against that of SNB-1 synaptobrevin (Figure 5A). This revealed a general trend whereby mutations that increased RAB-3 punctal fluorescence also tended to increase SNB-1 synaptobrevin punctal fluorescence (*R* = 0.59, *p* = 0.17), although this correlation was not significant. Three mutants, *egl-30* Gα_q_, *unc-11* AP180 and *aex-3* RabGEF, were outliers in this plot (Figure 5A, labeled in yellow). When these outliers were excluded, the correlation between RAB-3 and SNB-1 synaptobrevin became significant (*R* = 0.66, *p* = 0.016). Thus, across many conditions (22/25 mutants examined), SNB-1 and RAB-3 synaptic abundance was coordinately regulated.

The three outliers in the SNB-1 versus RAB-3 plot (Figure 5A) identify specific circumstances in which RAB-3 and SNB-1 were differentially regulated. The *aex-3* mutant lacks the GEF responsible for activating RAB-3 [38], and consequently would be expected to have a disproportionately stronger effect on RAB-3, compared to SNB-1. Disrupting *egl-30* Gα_q_ also caused a significantly greater decrease in RAB-3 punctal fluorescence than was observed for SNB-1 synaptobrevin (Figure 5A–C). This result suggests that *egl-30* Gα_q_ regulates the presynaptic levels of RAB-3 separately from SNB-1 synaptobrevin. Consistent with this idea, the protein localization profiles of *rab-3* and *egl-30* Gα_q_ mutants were significantly correlated (Figure 3A) (*R* = 0.55, *p* = 0.038), suggesting that these two mutations disrupt one or more processes in common. Thus, RAB-3 may be responsive to extracellular signals that couple to *egl-30* Gα_q_.

*unc-11* AP180 mutants had a disproportionately larger increase in the punctal fluorescence of RAB-3 compared to SNB-1 synaptobrevin (Figure 5A–C). *unc-11* AP180 mutants exhibit a specific defect in the endocytic recycling of SNB-1 synaptobrevin from the plasma membrane to SVs [13],[39]. Because SVs lacking SNB-1 synaptobrevin are predicted to be defective in exocytosis, the increase in RAB-3 punctal fluorescence in *unc-11* AP180 mutants (Figure 5B) may reflect the accumulation of defective SVs that contain insufficient amounts of SNB-1 synaptobrevin to undergo efficient exocytosis. Consistent with this hypothesis, increased RAB-3 punctal fluorescence was also observed in *snb-1* synaptobrevin mutants (Figure 5B). Moreover, the phenotypic profile of *unc-11* AP180 mutants clustered robustly with that of *snb-1* synaptobrevin mutants (Figure 2A). Taken together, these results suggest that the v-SNARE SNB-1 affects the recruitment of RAB-3 to presynaptic elements.

### Comparing Proteins that Act at Different Stages of the SV Cycle

SV precursors are transported to synapses by anterograde transport [40]. SYD-2 α-Liprin promotes anterograde transport of SV precursors to nerve terminals, while RAB-3 has been proposed to promote synaptic targeting of SVs, perhaps by mediating tethering of SV to active zone components [41],[42]. The puncta fluorescence for SYD-2 and RAB-3 were anti-correlated in our data set (Figure 3C). These results could indicate that RAB-3-mediated tethering negatively regulates SYD-2 α-Liprin-mediated SV transport. Consistent with this idea, SYD-2 α-Liprin punctal fluorescence was significantly increased in *rab-3* mutants (Figure 5D). The increased SYD-2 α-Liprin fluorescence was not observed in other exocytosis mutants (e.g. *unc-13* and *unc-18* nSec1 mutants) (Figure 5D). Taken together, these results suggest that RAB-3 activity somehow negatively regulates synaptic targeting of SYD-2 α-Liprin.

During the SV cycle, SVs undergo fusion with the plasma membrane to release neurotransmitters and are recycled locally by endocytosis. We examined several proteins that associate with SVs at various points during the SV cycle. SNB-1 synaptobrevin is a v-SNARE protein required for SV exocytosis. RAB-3 is a GTPase that reversibly associates with SVs in a manner that depends upon its bound nucleotide [43]. SNN-1 Synapsin has been proposed to associate with the reserve pool of SVs, mediating association of this pool of vesicles with F-actin [44]. APT-4 α2-adaptin associates with clathrin-coated vesicles, promoting recycling of SVs following fusion [45]. ITSN-1 Intersectin associates with both the cytoskeleton and components of the endocytic machinery to promote endocytosis [46]–[50] and is localized to presynaptic endocytic sites [48],[49]. These SV-associated proteins are thought to regulate different aspects of the SV cycle; consequently, one would expect that the abundance of these proteins would be differentially affected when specific steps of the SV cycle are disrupted. Our results are largely consistent with this idea.

The abundance of a protein associated with endocytic intermediates (APT-4 α2 adaptin), was negatively correlated with one associated with the pool of SNB-1 synaptobrevin positive vesicles (Figure 3C). The simplest interpretation of this result is that the size of the SNB-1 synaptobrevin positive pool of SVs is anti-correlated with the ongoing rate of secretion. When secretion rates are high this SNB-1 synaptobrevin positive SV pool is reduced whereas the converse change occurs when secretion rates are low. Similarly, high secretion rates would be expected to result in increased abundance of endocytic intermediates (labeled by APT-4 α2-adaptin and ITSN-1 intersectin) and newly recycled SVs.

The pattern of SNN-1 synapsin abundance in our mutant panel was anti-correlated with that of exocytic proteins SNB-1 synaptobrevin and RAB-3 (Figure 3C). Moreover, the SNN-1 synapsin pattern was most similar to that observed for the endocytic proteins APT-4 α2-adaptin and ITSN-1 intersectin (Figure S3). These relationships suggest that at the *C. elegans* NMJ, SNN-1 synapsin primarily associates with vesicles as they transit from the recycling endocytic intermediates, however this association is not maintained in the pool of SVs labeled by SNB-1 synaptobrevin and RAB-3. This result is consistent with prior studies showing that lamprey Synapsin-1 primarily associates with SVs distal from the active zone at rest and with peri-synaptic zones where endocytic recycling occurs during stimulation [44].

### Comparing SV and DCV Proteins

Neuropeptides and classical neurotransmitters are secreted by a similar calcium-dependent mechanism; however, the detailed mechanisms by which neuropeptides are synthesized and packaged into vesicles are quite distinct. Neuropeptides are initially synthesized as large proproteins that are packaged into dense core vesicle (DCV) precursors in the trans golgi network. Classical neurotransmitters are packaged in small clear synaptic vesicles (SV) that are clustered near release sites whereas large DCVs filled with neuropeptides are not restricted to nerve terminals. Moreover, exocytosis of DCVs can occur from both axons and dendrites. Different patterns of activity are typically required for evoking secretion of SVs versus DCVs, with higher frequencies or amplitudes required for the latter [51]. Different populations of DCVs within the same cell can contain different neuropeptides [52]. Here we focused on DCVs responsible for secreting an insulin/IGF family member, INS-22.

**Figure 6:**
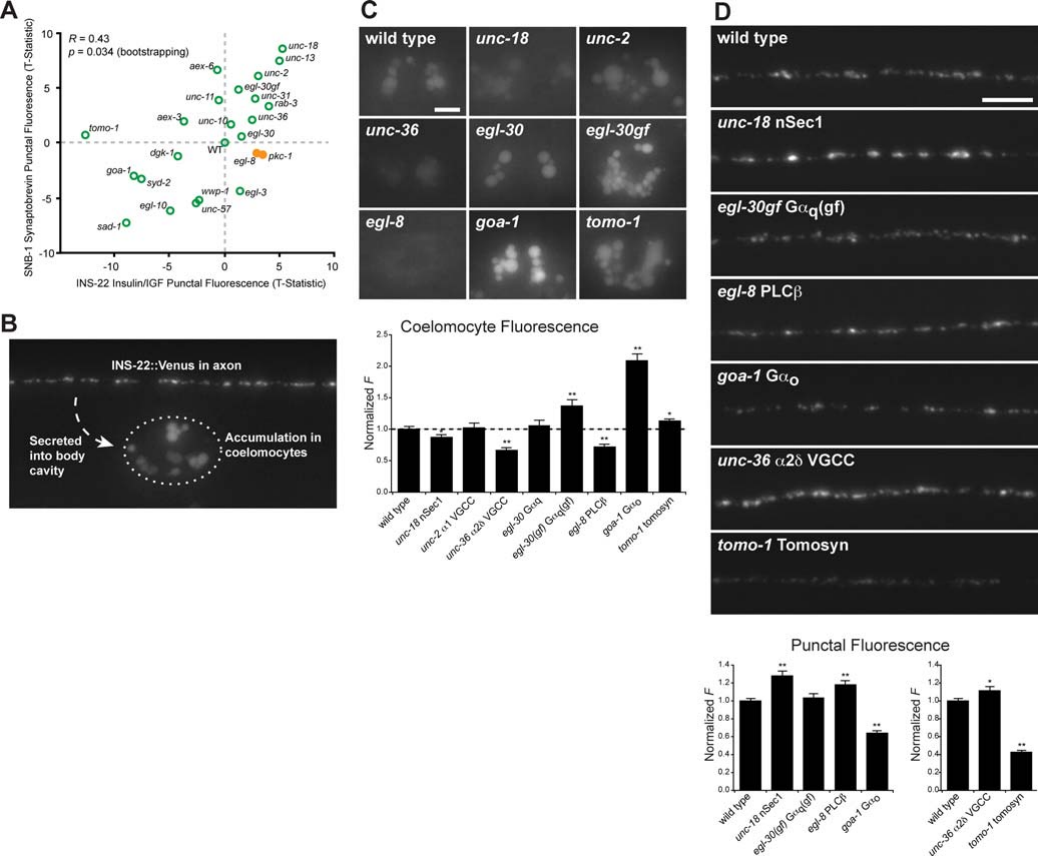
Analysis of DCV accumulation in axons and insulin/IGF secretion. (A) XY plot comparing changes in the punctal fluorescence of SNB-1 synaptobrevin and INS-22 insulin/IGF. Two mutants with more prominent increases in INS-22 insulin/IGF than SNB-1 synaptobrevin are shown as solid orange circles. (B) Secreted INS-22 insulin/IGF expressed in motorneurons accumulates in coelomocytes. (C) Images of INS-22 insulin/IGF accumulation in coelomocytes in wild type and mutant animals. Scale bar, 5 µm. Below is shown the quantification of coelomocyte INS-22 insulin/IGF fluorescence. (D) Images of axonal INS-22 insulin/IGF in wild type animals and mutants with altered INS-22 insulin/IGF coelomocyte fluorescence. Scale bar, 5 µm. Below is shown the quantification of INS-22 insulin/IGF punctal fluorescence. In (C–D), * indicates *p*<0.05, ** indicates *p*<0.01, (Student's T-Test compared to wild type). All error bars are ±SEM. Separate charts indicate data from separate sets of experiments.

To further characterize how classical neurotransmitters and neuropeptides are differentially regulated, we compared the protein localization profiles for SNB-1 synaptobrevin (a SV marker) and INS-22 insulin/IGF (a DCV marker) (Figure 6A). We previously showed that quantitative analysis of SNB-1 synaptobrevin and INS-22 insulin/IGF fluorescence in axons can be used as steady-state markers to assess the relative rates of SV and Insulin/IGF secretion respectively [13],[53]. The punctal fluorescence of INS-22 insulin/IGF and SNB-1 synaptobrevin were positively correlated in our mutant panel (*R* = 0.43, *p* = 0.03) (Figure 6A), suggesting that SV and INS-22 insulin/IGF secretion were coordinately regulated across these conditions. Many genes were required for both SV and INS-22 insulin/IGF secretion. For example, we found that both SVs and INS-22 insulin/IGF accumulated in exocytosis mutants, *unc-13* and *unc-18* nSec1 (Figure 6A).

Despite the overall positive correlation, there were some notable exceptions to this trend. For example, a mutation in the endocytic gene *unc-57* endophilin A strongly affected SNB-1 synaptobrevin punctal fluorescence (30% decrease, *p* = 1.2×10^−7^) but had a relatively weaker effect on INS-22 insulin/IGF fluorescence (14% decrease, *p* = 0.011) (Figure 6A, Table S2). This difference was expected since maintenance of the SV pool is mediated by local endocytic recycling at synapses, whereas maintenance of the DCV pool is mediated by anterograde transport from the golgi. SNB-1 synaptobrevin punctal fluorescence was affected by both *rab-3* and *aex-6* Rab27 mutations (Figure 6A, Table S2), consistent with prior studies showing that SV exocytosis was decreased in these mutants [54]. In contrast, INS-22 insulin/IGF fluorescence was increased in *rab-3* but not in *aex-6* Rab27 mutants (Figure 6A, Table S2). These results suggest RAB-3 plays a more prominent role than AEX-6 Rab27 in regulating INS-22 insulin/IGF transport or secretion.

Two mutations resulted in increased INS-22 insulin/IGF fluorescence while having little effect on SNB-1 synaptobrevin fluorescence (Figure 6A, yellow circles). One mutant corresponds to *pkc-1* protein kinase C η/ε (PKCη/ε), which regulates DCV secretion but not SV secretion [53]. The other corresponds to *egl-8* phospholipase Cβ (PLCβ). EGL-8 PLCβ is predicted to catalyze hydrolysis of phosphatidyl inositol (4,5) bisphosphate to produce DAG, an activator of PKC. This suggests that DAG produced by EGL-8 PLCβ may activate PKC-1 PKC η/ε to specifically regulate DCV secretion.

### Clusters of Genes that Regulate Insulin/IGF Secretion

Relatively few genes have been shown genetically to regulate insulin/IGF secretion in vivo. Clustering analysis of protein localization profiles identified two robust gene clusters predicted to be involved in DCV secretion (Figure 2A). The first cluster consisted of *unc-31* CAPS and *unc-36* α2δ subunit of a voltage-gated Ca^2+^ channel (α2δ VGCC) [55],[56]. *unc-31* CAPS is a multi-domain protein that has been previously implicated in DCV exocytosis in several systems [53], [55], [57]–[60]. The second cluster consisted of genes in the *egl-30* Gα_q_ pathway, including *egl-30* Gα_q_, *egl-8* PLCβ and *pkc-1* PKCη/ε [20],[53],[61],[62]. A major determinant for both of these clusters was a significant increase in the punctal fluorescence of INS-22 insulin/IGF (Figure 2B), suggesting that mutants in these clusters were defective in DCV exocytosis.

To verify that the genes in these clusters are required for INS-22 insulin/IGF secretion from DCVs, we measured secretion of INS-22 insulin/IGF from neurons in the corresponding mutants by quantitating steady-state fluorescence in coelomocytes. Coelomocytes are scavenger cells that take up secreted proteins. Secreted fluorescently tagged neuropeptides are endocytosed by coelomocytes, where they accumulate within endolysosomal organelles, which can be visualized as large internal fluorescent patches (Figure 6B) [53],[60]. Because the genes tested in this study are not expressed in the coelomocytes [14], [15], [22], [61]–[65] and do not appear to affect general endocytic traffic [66], the accumulation of INS-22 insulin/IGF in coelomocytes is therefore a measure of its secretion from DCVs in these mutants.

*unc-36* α2δ VGCC and *egl-8* PLCβ mutants both showed significant reductions in INS-22 insulin/IGF fluorescence in coelomocytes (Figure 6C), similar to the reductions previously observed for *pkc-1* PKC η/ε and *unc-31* CAPS [53],[60]. Moreover, both *unc-36* α2δ VGCC and *egl-8* PLCβ mutants showed accumulation of INS-22 insulin/IGF fluorescence in axons, indicating that reduced secretion was not due to reduced neuropeptide synthesis (Figure 6D). The clustering analysis, together with these results, strongly supports the idea that *unc-36* α2δ VGCC and *egl-8* PLCβ are required in some manner for INS-22 insulin/IGF secretion.

*unc-36* and *unc-2* encode the α2δ and α1 subunits of VGCCs respectively; mutants lacking either gene share a number of behavioral phenotypes in common [56],[63],[67],[68]. If UNC-36 α2δ VGCC and UNC-2 α1 VGCC function together in INS-22 insulin/IGF secretion, mutations in these subunits would be predicted to have similar protein localization profiles. Instead, we found that *unc-2* α1 VGCC mutants were not defective in INS-22 insulin/IGF secretion (Figure 6C), and *unc-2* α1 VGCC mutants did not cluster with *unc-36* α2δ VGCC (Figure 2A), suggesting that these VGCC subunits have different effects on presynaptic protein composition. These results suggest that INS-22 insulin/IGF secretion is promoted by a VGCC that requires the UNC-36 α2δ subunit but not the UNC-2 α1 subunit.

How are EGL-8 PLCβ and PKC-1 PKC η/ε activated to promote INS-22 insulin/IGF secretion? A putative activator of EGL-8 PLCβ is the alpha subunit of a heterotrimeric G protein EGL-30 Gα_q_. To determine if *egl-30* Gα_q_ also regulates INS-22 insulin/IGF secretion, we tested a partial loss-of-function allele of *egl-30* Gα_q_ because the null mutant is inviable [62]. INS-22 insulin/IGF coelomocyte fluorescence in these *egl-30* Gα_q_ mutants was indistinguishable from wild type controls (Figure 6C). It is possible that our assay was not sensitive enough to detect the subtler phenotype in the partial loss-of-function *egl-30* Gα_q_ mutant used. To further address whether *egl-30* Gα_q_ signaling is important for INS-22 insulin/IGF secretion, we examined an *egl-30(gf)* constitutively active Gα_q_ mutant [18]. We detected increased coelomocyte INS-22 insulin/IGF fluorescence in *egl-30(gf)* mutants (Figure 6C), suggesting INS-22 insulin/IGF secretion can be stimulated by *egl-30* Gα_q_ activity. Nevertheless, we cannot rule out the possibility that *egl-8* PLCβ may be regulated in an *egl-30* Gα_q_-independent manner.

Our analysis also identified negative regulators of INS-22 insulin/IGF secretion. *goa-1* Gα_o_ and *tomo-1* tomosyn have been shown to negatively regulate SV exocytosis in *C. elegans* [15],[19],[69]. In *goa-1* Gα_o_ mutants, INS-22 insulin/IGF secretion increased dramatically (Figure 6C). A corresponding reduction in INS-22 insulin/IGF fluorescence was observed in axons (Figure 6D), consistent with a depletion of DCVs containing INS-22 insulin/IGF due to excess release. Similar results were observed in *tomo-1* tomosyn mutants (Figure 6C–D), in agreement with another recent study [16]. These results indicate that *goa-1* Gα_o_ and *tomo-1* tomosyn inhibit INS-22 insulin/IGF secretion.

### Genes that Regulate Insulin/IGF Secretion also Regulate Lifespan in an Insulin/IGF-Dependent Manner

How do changes in protein localization profiles impact the physiology of the whole animal? In *C. elegans*, disruption of insulin/IGF signaling results in increased longevity [8]. To determine whether the genes identified in this study that regulate the secretion of one insulin/IGF (INS-22) also affect lifespan, we tested the corresponding mutants for changes in lifespan.

**Figure 7:**
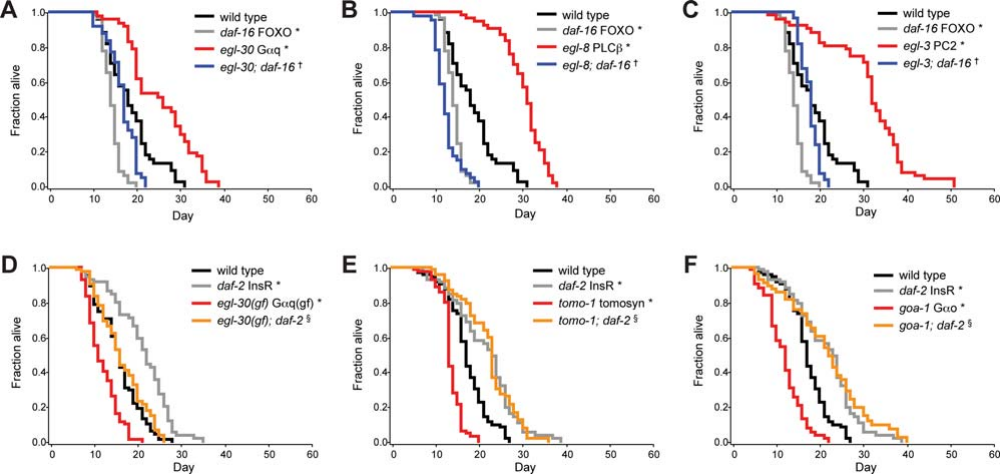
Insulin/IGF secretion mutants show lifespan phenotypes. (A–F) Survival curves with indicated genotypes. * indicates significantly different lifespan from wild type (*p*<0.0001), † indicates significant suppression by *daf-16* FOXO (*p*<0.0001), § indicates significant suppression by *daf-*2 InsR (*p*<0.0001), (Log Rank Test).

*egl-30* Gα_q_ and *egl-8* PLCβ mutants were long-lived (Figure 7A–B). Furthermore, the increased longevity of these mutants was suppressed by a deletion of *daf-16* FOXO, a transcription factor that is activated when insulin/IGF signaling is reduced (Figure 7A–B) [8]. Conversely, *egl-30(gf)* constitutively active Gα_q_ mutants were short-lived (Figure 7D). Because *egl-30(gf)* constitutively active Gα_q_ mutants exhibited increased INS-22 insulin/IGF secretion, they were predicted to have excess insulin/IGF signaling. Consistent with this prediction, the shortened lifespan of *egl-30(gf)* mutants was partially suppressed by a mutation in the *daf-2* insulin/IGF receptor (InsR) (Figure 7D). These results imply that the regulation of lifespan by the *egl-30* Gα_q_ pathway is bidirectional and requires InsR and FOXO signaling, further supporting a role for these genes in regulating insulin/IGF secretion.

The secretion of active insulin from mammalian cells requires processing by proprotein convertase 2 (PC2) [70], suggesting that *C. elegans egl-3* PC2 might also be involved in insulin/IGF processing [71]. Since the phenotypic profile of *egl-3* PC2 was significantly correlated with *egl-30* Gα_q_ and *egl-8* PLCβ (Figures 3A, S2), we examined *egl-3* mutants for alterations in lifespan. In agreement with a previous RNAi study [72], *egl-3* PC2 mutants are long-lived in a *daf-16* FOXO dependent manner (Figure 7C). This raises the possibility that *egl-3* PC2 might be involved in insulin/IGF processing, although we cannot rule out roles for *egl-3* PC2 in processing other neuropeptides that regulate *C. elegans* lifespan.

*tomo-1* tomosyn and *goa-1* Gα_o_ mutants had increased INS-22 insulin/IGF secretion and were short-lived (Figure 7E–F). These reductions in lifespan required normal insulin/IGF signaling, as disrupting *daf-2* InsR in these mutant backgrounds suppressed their short-lived phenotype (Figure 7E–F). These results further support the idea that *tomo-1* tomosyn and *goa-1* Gα_o_ inhibit insulin/IGF secretion. Together our findings indicate that lifespan can be regulated bidirectionally by genes that control insulin/IGF secretion. These results also provide an example where changes in presynaptic protein localization profiles can be mechanistically associated with changes in the physiology of the animal.

## Discussion

Here we present a systematic strategy for dissecting the structure and function of a subcellular specialization. We examined how mutations disrupting neurotransmission affected a set of presynaptic proteins at the *C. elegans* NMJ. This analysis led to several principal findings. First, the protein composition of active zones and SVs vary in different mutant backgrounds, suggesting that these structures are plastic. Second, we identified new genes involved in DCV-mediated secretion of insulin/IGF hormones. Third, several of these genes regulate the lifespan of *C. elegans* in a manner dependent on the endogenous insulin/IGF neuroendocrine signaling pathway, indicating the physiological significance of regulating insulin/IGF secretion. Fourth, our results demonstrate the feasibility and utility of this approach to understanding the in vivo structure and function of cellular compartments in intact multicellular animals.

Before discussing these results, it is worth considering the limitations of our strategy. First, although high content imaging screens are becoming more routine, high-resolution quantitative fluorescence microscopy remains somewhat labor intensive. Second, to limit the amount of imaging involved in our analysis, we analyzed only a single allele for each mutant in our panel. Although we used well-characterized alleles and strains, it remains possible that some of the effects reported here are caused by other mutations in these strains. Third, we only examined one transgene for each marker; consequently, some of the protein localization phenotypes reported here may be dependent on transgene expression-level or on position effects. Fourth, some of the phenotypes we observed could be due to non-autonomous effects from other neurons; for example, neuropeptides regulate neurotransmission at the NMJ and could act from distant neurons [73]. Fifth, we only characterized a single DCV marker (INS-22); therefore, it is unclear whether similar effects would be observed for other classes of neuropeptides. Sixth, all of our markers were imaged in a single class of cholinergic motor neurons (DA neuron). It is likely that the mutant panel might have distinct effects on other neurons and synapses. For example, prior studies have shown that the synaptogenic molecule SYD-2 α-Liprin has very distinct effects on synapses formed by different classes of neurons [30],[31],[74]. Despite these limitations, our results suggest several new insights into how presynaptic protein composition is regulated.

### Plasticity of Protein Composition in Subcellular Compartments

Regulating the levels of a single presynaptic protein can be crucial in tuning neurotransmitter secretion. For example, increasing the levels of UNC-10 or its ortholog RIM1α can lead to increased neurotransmitter secretion [35],[75]. Several layers of regulation ensure that appropriate levels of UNC-10 or RIM1α are present at presynaptic specializations, including *syd-2* α-Liprin-dependent and independent means of UNC-10 RIM1α recruitment [33; this work], as well as ubiquitin-mediated degradation of RIM1α [75].

We analyzed multiple synaptic markers, allowing us to detect trends and correlations not possible from studying individual markers. One theme that emerged was that markers localized to the same synaptic compartments could differ in their response to disruption of presynaptic function. For example, changes in the punctal fluorescence of active zone markers UNC-10 RIM1α and SYD-2 α-Liprin did not correlate when examined across a panel of neurotransmission mutants, suggesting that additional factors besides SYD-2 α-Liprin can exert a significant impact on UNC-10 RIM1α abundance. This result could represent changes in the ability or specificity of the proteins to localize to certain subcellular structures. Alternatively, this result may reflect altered rates of protein synthesis or turnover. Together, our data suggests that the composition of the active zone can be altered in response to changes in presynaptic function. The ability to independently regulate different components of the active zone could provide a mechanism to fine-tune neurotransmission.

Detailed proteomic studies have revealed the protein components of SVs [76], some of which were studied here. We show that compositional changes among SV proteins can be observed when specific aspects of synaptic function are perturbed. Because SVs exist in functionally distinct pools that have been proposed to contain different molecular constituents [5], the compositional changes we observed might reflect shifts in the relative abundance of SV pools. For example, the abundance of SNN-1 synapsin was negatively correlated with both RAB-3 and SNB-1 synaptobrevin, possibly indicative of changes in their association with intermediates during the SV cycle. These relationships are consistent with the idea that different sets of proteins transiently associate with SVs as they traverse through different steps in the exocytosis/endocytosis cycle.

### New Genes Involved in Insulin/IGF Secretion from DCVs

Most work on DCV secretion has focused on cultured neurosecretory cells; less is known about the cell biology of DCV secretion in neurons of intact animals. We found a role for *unc-36* α2δ VGCC in DCV secretion, which had not been previously implicated in this process. Prior work in *Drosophila* revealed that an α2δ VGCC subunit encoded by *straitjacket* is required for SV exocytosis [77],[78]. Thus, it is possible that α2δ VGCC subunits are involved in both SV and DCV secretion.

The *unc-2* α1 VGCC subunit [67] was a candidate for functioning in the same channel as *unc-36* α2δ VGCC because they shared many behavioral and developmental phenotypes [56],[63],[68]. However, *unc-2* α1 VGCC did not co-cluster with either *unc-36* α2δ VGCC or *unc-31* CAPS; furthermore, *unc-2* α1 VGCC mutants did not show a detectable INS-22 insulin/IGF secretion defect. Thus, while *unc-36* α2δ VGCC and *unc-2* α1 VGCC may act together for certain processes, they may also participate in the formation of distinct channels, perhaps as a mechanism for increasing channel diversity in the nervous system. Our analysis was able to dissect the functions of these two VGCC subunits by separating them into two clusters. In *Drosophila*, *straitjacket* α2δ VGCC is required for proper localization of the *cacophony* α1 VGCC subunit required for SV secretion [77],[78]. This raises the possibility that UNC-36 α2δ VGCC might also localize another, presently unidentified, α1 VGCC subunit involved in DCV secretion.

*egl-8* PLCβ was also identified as a new positive regulator of INS-22 insulin/IGF secretion. Our previous work implicated *pkc-1* PKCη/ε in DCV exocytosis and showed that an activated *pkc-1* PKCη/ε mutation was epistatic to *egl-8* PLCβ; this argued that *pkc-1* PKCη/ε acts downstream of *egl-8* PLCβ [53]. Here we showed that these two genes clustered together, indicating they have a similar spectrum of phenotypes, and are thus likely to act within the same pathway, rather than in parallel pathways. One model supported by our results is that EGL-8 PLCβ catalyzes the formation of a second messenger, DAG, to activate PKC-1 PKCη/ε, which in turn promotes DCV exocytosis [53]. This pathway appeared to be specific to DCV rather than SV secretion and may thus serve to regulate the types of transmitters secreted by a neuron.

We identified *goa-1* Gα_o_ as a new negative regulator of insulin/IGF secretion. *goa-1* Gα_o_ also negatively regulates SV exocytosis in the same set of neurons [17],[20],[21],[69], consistent with a decrease in the punctal fluorescence of SNB-1 synaptobrevin in *goa-1* Gα_o_ mutants (Table S2). Together, this suggests that *goa-1* may function as a regulator of secretion from both SVs and DCVs. The effect of GOA-1 Gα_o_ on active zone components such as SYD-2 α-Liprin, UNC-10 RIM1α and UNC-13 likely contributes to its role to regulating SV secretion. Since DCV secretion does not occur at active zones [58], GOA-1 Gα_o_ likely regulates DCV secretion through effectors in other compartments. Whether the same pools of GOA-1 Gα_o_ act to coordinate SV and DCV secretion or are regulated distinctly for each of these functions also remains to be determined.

### Modulating Lifespan by Regulating Insulin/IGF Secretion

Aging is modulated by a conserved insulin/IGF signaling pathway in *C. elegans* and other species [8]. Whereas much attention has been focused on the pathways and effectors downstream of insulin/IGF receptor in the regulation of lifespan, little is known about how insulin/IGF secretion is regulated to initiate this process. Mutations that disrupt the core DCV exocytic machinery lead to increased longevity [79], but the pathways that regulate insulin/IGF secretion in *C. elegans* lifespan control were previously unknown. Here, we identified G-protein and second messenger pathways that modulate insulin/IGF secretion and control *C. elegans* lifespan in an insulin/IGF signaling-dependent manner. Our results suggest that the bidirectional regulation of insulin/IGF secretion by these pathways are endogenous determinants of *C. elegans* lifespan.

Elegant studies have indicated that communication between different tissues is required for proper regulation of lifespan [8]. The nervous system is the predominant locus of insulin/IGF expression in *C. elegans* [80] and may function as a signaling center in this process. Because the molecules identified here as regulators of insulin/IGF secretion are expressed throughout the *C. elegans* nervous system, they are likely to act as general rather than cell-specific factors. In this context, it is particularly interesting to identify signaling molecules such as G-proteins as regulators of insulin/IGF secretion. Since G-proteins mediate responses to extracellular signals, they provide an attractive mechanism for coupling changes in neuronal signaling to changes in lifespan.

### A Systematic Genetic Approach to Analyzing Subcellular Compartments

The proliferation of genomic and proteomic studies has provided substantial knowledge of cellular organization and function. Addressing how the genome regulates the proteome is a logical next step to link these two types of information. Our results demonstrate that even analyzing the relationship between a small, focused subset of genes and proteins can yield new and detailed information about a specific subcellular specialization. Thus, connecting gene function to protein localization can serve as a platform to understand detailed and global properties of subcellular compartments, the proteins that inhabit them and the genes that regulate these proteins. With advances in automated microscopy, we anticipate that extending our approach and analytical techniques to additional subcellular compartments across many genetically tractable systems will yield a wealth of biological information.

## Materials and Methods

### Strains and Genetics

All strains were cultivated at 20°C using standard methods. The following mutations or transgenes were used in this analysis: *unc-18(md1088)*, *unc-13(s69)*, *unc-2(lj1)*, *unc-31(e928)*, *aex-3(js815)*, *aex-6(sa24)*, *egl-10(n692)*, *unc-36(e251)*, *egl-30(ad806)*, *egl-30(js126gf)*, *unc-10(e102)*, *egl-3(nr2090)*, *egl-8(sa47)*, *tomo-1(nu468)*, *rab-3(js49)*, *unc-26(s1710)*, *unc-11(e47)*, *sad-1(ky289)*, *wwp-1(ok1102)*, *dgk-1(nu62)*, *goa-1(sa734)*, *unc-57(e406)*, *pkc-1(nj3)*, *syd-2(ju37)*, *snb-1(md247)*, *daf-2(e1368)*, *daf-16(mu86)*, *nuIs152[ttx-3::mRFP, Punc-129::GFP::snb-1]II*, *nuIs159[ttx-3::mRFP, Punc-129::GFP::syd-2]III*, *nuIs163[myo-2::GFP, Punc-129::snn-1::Venus]II*, *nuIs165[myo-2::GFP, Punc-129::unc-10::GFP]II*, *nuIs168[myo-2::GFP, Punc-129::Venus::rab-3]IV*, *nuIs169[myo-2::GFP, Punc-129::gelsolin::Venus]III; nuIs184[myo-2::GFP, Punc-129::apt-4::GFP]X*, *nuIs190 X and nuIs195[myo-2::GFP, Punc-129::ins-22::Venus]IV*, *nuIs214[myo-2::GFP, Punc-129::itsn-1::GFP]III*. All integrated transgenes were outcrossed 10 times to wild type N2. For each marker, we selected one out of several integrated transgenes that displayed the most consistent and representative pattern of synaptic localization. Strains were genotyped by sequencing or PCR where appropriate. All mutants are described in www.wormbase.org.

### Molecular Biology

All GFP/YFP-labeled markers were expressed in the DA class of motorneurons under the *Punc-129* promoter. All plasmids used to label presynaptic compartments are derivatives of pPD49.26 containing an SphI/BamHI *unc-129* promoter fragment. All constructs were sequenced as to ensure that they contained wild type sequences. For the following constructs, all GFP or Venus fragments were cloned in-frame to the synaptic genes and the fusions were subcloned as NheI/KpnI fragments: KP#1283 *Punc-129::GFP::snb-1* [11]; KP#1483 *Punc-129::GFP::syd-2* (gift of D. Simon); pDS171 *Punc-129::snn-1::*Venus (*snn-1* cDNA fragment was used); pDS203 *Punc-129::unc-10::GFP* [*unc-10::GFP* (gift of D. Simon) was subcloned as an NheI/KpnI fragment]; pDS165 *Punc-129::Venus::rab-3* (the 6b isoform of *rab-3 cDNA* was used, and the 5′ end of *rab-3* contains the attL1 gateway site); pDS233 *Punc-129::itsn-1::GFP* (*itsn-1* cDNA::GFP was a gift from J. Bai); and pDS210 *Punc-129::apt-4::GFP* (*apt-4* cDNA was used and is flanked by gateway attL1 and R1 sites).

For the following constructs, entry clones from the ORFeome project corresponding to the gene used was cloned into the destination vector KP#1284 [11] using the gateway strategy with LR clonase (Invitrogen): pDS178 *Punc-129::gelsolin::Venus* and KP#1496 *Punc-129::ins-22::Venus*.

KP#708 *Pttx-3::mRFP* or pPD118.33 *Pmyo-2::GFP* were used as transgenic markers. Presynaptic marker constructs were injected at 10–25ng/ul, and transgenic markers were injected at 50 ng/µl for KP#708 and 10 ng/µl for pPD118.33.

### Microscopy and Image Analysis

Young adult animals were paralyzed using 30 mg/ml BDM (Sigma) and mounted on 2% agarose pads for imaging. Images were acquired on a Zeiss Axiovert 100 microscope using an Olympus Planapo 100× objective (NA = 1.4) and an ORCA 100 CCD (Hamamatsu) controlled by Metamorph 4.5 software (Universal Imaging/Molecular Devices). Animals were imaged as previously described [11],[53]. For dorsal cord imaging, ∼30 dorsally oriented animals per genotype were imaged near the posterior gonad bend. A maximum intensity projection was obtained from image stacks of the dorsal axon, the axon was traced in Metamorph 4.5 and traces containing fluorescence intensity along the axon were analyzed in custom software written in Igor Pro (Wavemetrics) as previously described [12],[13]. For coelomocyte imaging, ∼20–60 laterally oriented animals where the coelomocyte was not obscured by other tissues were imaged. A maximum intensity projection was obtained from image stacks of the coelomocyte and the mean fluorescence within each vesicle in the coelomocyte were recorded in Metamorph 7; these values were analyzed in Igor Pro to obtain mean coelomocyte fluorescence for each genotype as previously described [53]. All fluorescence values in this study were normalized to the fluorescence of 0.5 µm FluoSphere beads (Molecular Probes) captured during each imaging session to provide a standard for comparing absolute fluorescence levels between animals from different sessions. Some *nuIs152* data for this analysis was obtained from Sieburth et. al., [11] and McEwen et al., [19].

Under the conditions used for imaging, we determined that UNC-10::GFP, GFP::SYD-2 and APT-4::GFP were exclusively or predominantly localized to synaptic puncta, as we could detect little or no difference between their axonal fluorescence and the autofluorescence of *C. elegans*. For these markers, we excluded the axonal fluorescence in our analysis. Similarly, we excluded the FWHM for diffraction limited or near-diffraction limited markers (UNC-10:GFP, GFP::SYD-2, Gelsolin::Venus, SNN-1::Venus) where the physical limitations of conventional light microscopy might prevent an accurate estimate of these values.

### Correlation, Clustering, and Bootstrapping Analysis

The Student's T-statistic was used as a numerical score to represent the difference between wild type and mutant animals for each parameter of each marker (Table S3); this created a numerical profile of phenotypes or marker behavior for further analysis. Correlation analysis was performed in Igor Pro (Wavemetrics). Hierarchical clustering was performed with Cluster 3.0 [2],[81]; the 24 clustering methods used were all combinations of 6 distance measures (uncentered correlation, centered correlation, Spearman's Rank, Kendall's Tau, City-Block and Euclidean distance) and 4 linkage methods (maximum, minimum, centroid and average) (Table S1).

We identified several robust clusters based on unbiased, stringent criteria, requiring these clusters be detected in 12 or more out of 24 different clustering strategies used to analyze this dataset. Also, the phenotypic profiles in these clusters had to be significantly correlated (*p*<0.05 with Bonferroni Correction). See Supporting Information in Text S1 for additional criteria. Using the Pearson's Correlation as a distance measure reproduced all the robust clusters identified in our dataset, justifying the use of this measure for comparing phenotypic and marker profiles.

The dendrogram and heat maps were visualized with JavaTree [82]. Custom software written in Igor Pro (Wavemetrics) was used for all other clustering analysis, including the generation of the numerical scores for clustering, counting the number of times a cluster of genes appeared across the 24 combinations of clustering algorithms and calculating the importance of each parameter.

For each cluster, we calculated a score indicating how each parameter contributed to the similarity among genes in that cluster based on how removal of the parameter from the analysis affected the similarity between phenotypic profiles within that cluster. For a given cluster, parameters that capture the majority of the contributing phenotypes (i.e. those that comprise top 95% of the cumulative contributing score) were deemed as important to that cluster (see Supporting Information in Text S1). To confirm the importance of these parameters, we repeated the clustering analysis using only these parameters for each cluster. In each case, we identified the cluster of genes, often with better robustness (as determined by the number of clustering methods that gave rise to that cluster) despite the reduction in the number of parameters used (Figure S1).

To determine the significance of the correlation coefficients, we performed a bootstrapping analysis. For phenotypic correlation, we computed the correlation coefficients for 100,000 pairs of permutated phenotypic profiles, where each parameter in the profile was randomly drawn from the dataset. The resulting distribution of correlation coefficients allowed us to estimate how frequently a correlation coefficient would arise by chance alone. The significance of the correlation for the actual data was calculated as the fraction of correlation coefficients from the random permutations that gave a stronger score. Similar analyses were performed for the correlation between marker punctal fluorescence profiles.

### Lifespan Assays

Lifespan assays were performed essentially as previously described [83]. For *egl-30(gf)*, *goa-1* and *tomo-1* strains and controls, animals were transferred to a fresh plates each day during their fertile period to separate them from their progeny. For *egl-3*, *egl-30* and *egl-8* strains and controls, animals were assayed on plates containing 0.1mg/ml 5-fluorodexoyuridine (Sigma) to kill their progeny [84] and prevent premature death due to internal hatching of progeny in these egg-laying defective mutants. Statistical analysis of survival was performed with SPSS 11 (SPSS. Inc).

## Supporting Information

Figure S1 Influence of parameters in generating clusters. See Text S1 for details. (A) and (B) Plots of clustering robustness (occurrence of cluster in out of 24 clustering methods) and the cumulative fraction of the maximal CCS with inclusion of parameters in rank order. The cluster analyzed is indicated in each chart.
(0.55 MB PDF)

Figure S2 Comparison of phenotypic profiles between *egl-3* PC2 and genes involved *egl-30* Gα_q_ signaling. *P* values are calculated from bootstrapping analysis and indicated below each comparison.
(0.22 MB PDF)

Figure S3 Simulation of *ctrA401ts*. Significant correlations are highlighted as indicated by the legend.
(0.26 MB PDF)

Table S1 Clustering outcomes across multiple clustering methods.
(0.12 MB PDF)

Table S2 Quantitative imaging of presynaptic markers.
(0.77 MB PDF)

Table S3 Scores for clustering.
(0.05 MB PDF)

Text S1 Supporting information. Calculating the importance of each parameter to a cluster.
(0.16 MB PDF)
