# Supplementary figures and images for: Profiling Synaptic Proteins Identifies Regulators of Insulin Secretion and Lifespan
Source: PLoS Genet. 2008 Nov 28;4(11):e1000283. doi: 10.1371/journal.pgen.1000283 (PMC2582949; doi:10.1371/journal.pgen.1000283)

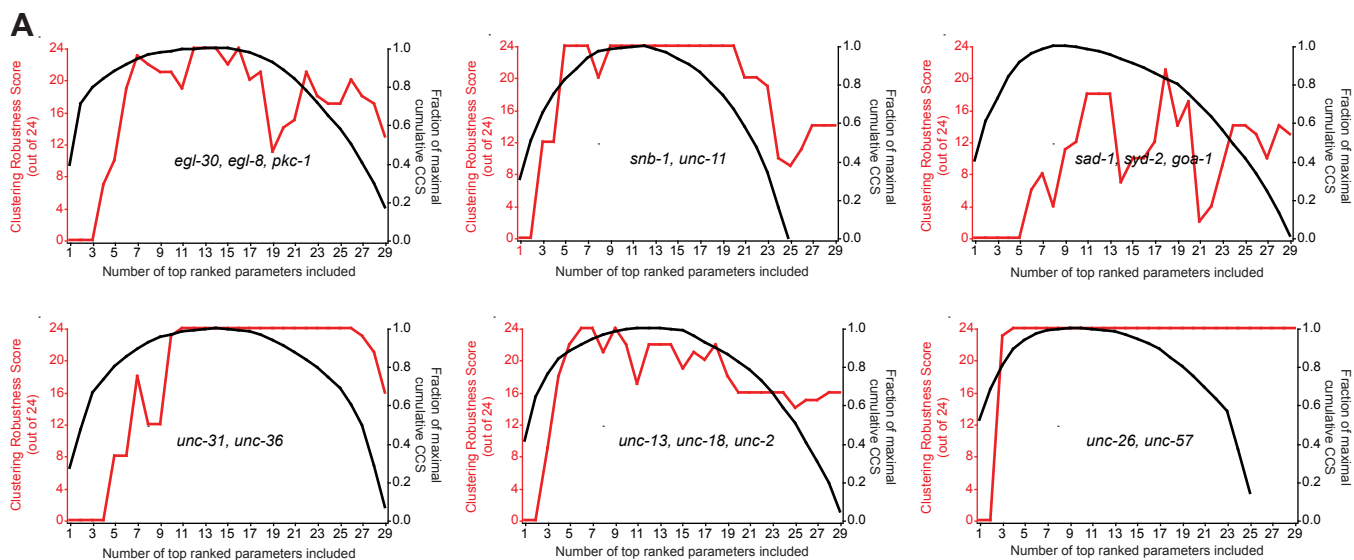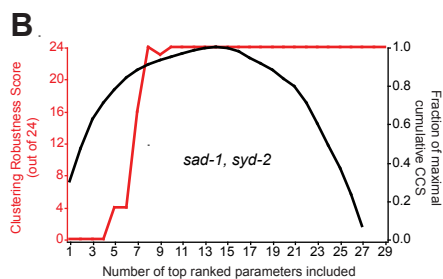

Supplement: Figure S1 — Influence of parameters in generating clusters. See Text S1 for details. (A) and (B) Plots of clustering robustness (occurrence of cluster in out of 24 clustering methods) and the cumulative fraction of the maximal CCS with inclusion of parameters in rank order. The cluster analyzed is indicated in each chart. (0.55 MB PDF) [file pgen.1000283.s001.pdf]

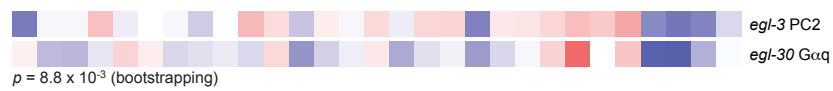

$p = 8.8 \times 10^{-3}$  (bootstrapping)

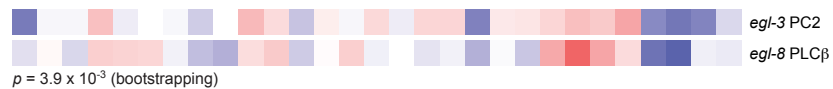

$p = 3.9 \times 10^{-3}$  (bootstrapping)

Supplement: Figure S2 — Comparison of phenotypic profiles between egl-3 PC2 and genes involved egl-30 Gαq signaling. P values are calculated from bootstrapping analysis and indicated below each comparison. (0.22 MB PDF) [file pgen.1000283.s002.pdf]
