# Supplementary material for: Profiling Synaptic Proteins Identifies Regulators of Insulin Secretion and Lifespan
Source: PLoS Genet. 2008 Nov 28;4(11):e1000283. doi: 10.1371/journal.pgen.1000283 (PMC2582949; doi:10.1371/journal.pgen.1000283)

Similarity to SNN-1 Punctal Fluorescence

Parameter	Correlation
APT-4	0.488196
ITSN-1	0.323971
UNC-10	-0.014186
Gelsolin	-0.0149626
SYD-2	-0.101501
INS-22	**-0.15571**
SNB-1	**-0.245176**
RAB-3	**-0.405198**

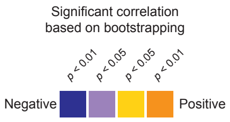

Supplement: Figure S3 — Simulation of ctrA401ts. Significant correlations are highlighted as indicated by the legend. (0.26 MB PDF) [file pgen.1000283.s003.pdf]
