# Supplementary material for: Profiling Synaptic Proteins Identifies Regulators of Insulin Secretion and Lifespan
Source: PLoS Genet. 2008 Nov 28;4(11):e1000283. doi: 10.1371/journal.pgen.1000283 (PMC2582949; doi:10.1371/journal.pgen.1000283)
Supplement: Table S1 — Clustering outcomes across multiple clustering methods. (0.12 MB PDF) [file pgen.1000283.s004.pdf]

Table S1 - Clustering outcomes across multiple clustering methods

Genes in Cluster	Clustering Method: Pearson's Correlation (uncentered)				Pearson's Correlation (centered)				Spearman's Rank Correlation				Kendall's Tau				Euclidean Distance				City Block Distance				score
	a	c	m	s	a	c	m	s	a	c	m	s	a	c	m	s	a	c	m	s	a	c	m	s	
sad-1 syd-2	■	■	■	■	■	■	■	■	■	■	■	■	■	■	■	■	■	■	■	■	■	■	■	■	**24**
unc-13 unc-18	■	■	■	■	■	■	■	■	■	■	■	■	■	■	■	■	■	■	■	■	■	■	■	■	**24**
unc-26 unc-57	■	■	■	■	■	■	■	■	■	■	■	■	■	■	■	■	■	■	■	■	■	■	■	■	**24**
aex-3 aex-6 dgk-1 egl-10 egl-3 egl-30 egl-30gf egl-8 goa-1 pkc-1 rab-3 sad-1 snb-1 syd-2 tomo-1 unc-10 unc-11 unc-13 unc-18 unc-2 unc-26 unc-31 unc-36 unc-57 wwp-1	■	■	■	■	■	■	■	■	■	■	■	■	■	■	■	■	■	■	■	■	■	■	■	■	**24**
egl-30 egl-8	■	■	■	■	■	■	■	■									■	■	■	■	■	■	■	■	**16**
unc-31 unc-36	■	■	■		■		■		■	■	■		■	■	■		■		■	■	■		■		**16**
unc-13 unc-18 unc-2	■	■	■	■	■	■	■	■	■	■	■	■	■	■	■	■									**16**
snb-1 unc-11	■	■	■	■	■	■	■	■									■		■		■	■	■	■	**14**
goa-1 sad-1 syd-2	■	■	■		■	■	■	■									■	■	■		■	■	■		**13**
egl-30 egl-8 pkc-1	■	■	■	■	■	■		■									■	■	■	■		■		■	**13**
goa-1 sad-1 syd-2 tomo-1	■	■	■		■	■	■										■	■	■		■		■		11
egl-3 egl-30 egl-8 pkc-1	■	■		■	■	■		■					■				■	■		■					10
aex-3 aex-6 dgk-1 egl-10 egl-3 egl-30 egl-30gf egl-8 goa-1 pkc-1 rab-3 sad-1 snb-1 syd-2 tomo-1 unc-10 unc-11 unc-13 unc-18 unc-2 unc-31 unc-36 wwp-1		■		■		■		■	■	■		■	■	■		■									10
egl-30gf rab-3 unc-13 unc-18 unc-2	■	■	■	■	■	■				■			■	■		■									10
dgk-1 egl-10 goa-1 sad-1 syd-2 tomo-1 wwp-1	■	■		■	■	■		■	■		■		■												9
rab-3 unc-13 unc-18 unc-2	■	■	■		■	■				■			■	■	■										9
egl-3 egl-30 egl-8							■			■		■	■	■	■	■							■		8
egl-30gf unc-2																	■	■	■	■	■	■	■	■	8
dgk-1 egl-10 goa-1 sad-1 syd-2									■	■	■	■	■	■	■	■									8
aex-6 unc-11									■	■	■	■	■	■	■	■									8
egl-3 egl-8									■	■	■	■	■	■	■	■									8
aex-6 unc-31 unc-36	■	■	■		■		■												■				■		7
aex-6 snb-1 unc-11									■	■	■		■	■	■	■									7
dgk-1 goa-1 sad-1 syd-2 tomo-1 wwp-1	■	■		■	■	■		■																	6
egl-3 egl-30 egl-8 pkc-1 wwp-1										■				■			■	■	■		■				6
aex-6 dgk-1 egl-3 egl-30 egl-8 pkc-1 unc-31 unc-36 wwp-1																	■			■	■	■		■	5
egl-3 egl-30 egl-8 pkc-1 unc-31 unc-36 wwp-1																	■	■		■	■			■	5
dgk-1 egl-3 egl-30 egl-8 pkc-1 unc-31 unc-36 wwp-1																	■	■			■	■		■	5
dgk-1 goa-1 sad-1 syd-2									■			■	■		■	■									5
egl-30gf unc-13 unc-18 unc-2				■				■	■			■				■									5
dgk-1 sad-1 syd-2									■			■	■		■	■									5
aex-6 dgk-1 egl-3 egl-30 egl-30gf egl-8 pkc-1 rab-3 unc-10 unc-2 unc-31 unc-36 wwp-1																	■	■			■	■		■	5
aex-6 egl-3 egl-30 egl-30gf egl-8 pkc-1 rab-3 unc-10 unc-13 unc-18 unc-2 unc-31 unc-36	■	■			■	■		■																	5
aex-3 aex-6 dgk-1 egl-10 egl-3 egl-30 egl-30gf egl-8 goa-1 pkc-1 rab-3 sad-1 syd-2 tomo-1 unc-10 unc-13 unc-18 unc-2 unc-31 unc-36 wwp-1	■		■		■		■	■																	5
egl-30gf rab-3 unc-10 unc-13 unc-18 unc-2					■	■				■				■											4
goa-1 sad-1 syd-2 tomo-1 wwp-1	■	■			■	■																			4
snb-1 unc-11 unc-26 unc-57	■		■		■		■																		4
aex-6 dgk-1 egl-3 egl-30 egl-30gf egl-8 pkc-1 rab-3 unc-2 unc-31 unc-36 wwp-1																		■			■	■		■	4
aex-3 dgk-1 egl-10 goa-1 sad-1 syd-2 tomo-1 wwp-1	■	■			■	■																			4
tomo-1 wwp-1									■		■		■		■										4
aex-3 unc-10											■		■		■										3
aex-3 aex-6 dgk-1 egl-3 egl-30 egl-30gf egl-8 pkc-1 rab-3 unc-10 unc-2 unc-31 unc-36 wwp-1																			■		■		■		3
aex-3 aex-6 dgk-1 egl-3 egl-30 egl-30gf egl-8 goa-1 pkc-1 rab-3 sad-1 snb-1 syd-2 tomo-1 unc-10 unc-11 unc-13 unc-18 unc-2 unc-26 unc-31 unc-36 unc-57 wwp-1																					■	■		■	3
egl-3 egl-30 egl-30gf egl-8 pkc-1 rab-3 unc-13 unc-18 unc-2 unc-31 unc-36								■					■		■										3
aex-3 dgk-1 egl-10 goa-1 sad-1 syd-2 tomo-1			■							■				■											3
egl-3 egl-30 egl-8 pkc-1 unc-36 wwp-1																		■				■		■	3
egl-3 egl-30 egl-8 wwp-1							■			■				■											3
aex-3 rab-3							■												■				■		3
aex-3 aex-6 dgk-1 egl-3 egl-30 egl-30gf egl-8 goa-1 pkc-1 rab-3 sad-1 syd-2 tomo-1 unc-10 unc-13 unc-18 unc-2 unc-26 unc-31 unc-36 unc-57 wwp-1																					■	■		■	3
egl-10 goa-1 sad-1 syd-2 tomo-1																	■		■				■		3
egl-10 sad-1 syd-2										■	■			■											3
dgk-1 egl-10 goa-1 sad-1 syd-2 wwp-1												■				■									2
aex-3 dgk-1 egl-10 goa-1 sad-1 syd-2										■				■											2
aex-6 egl-30gf rab-3 unc-10 unc-13 unc-18 unc-2 unc-31 unc-36		■			■																				2
egl-3 egl-8 pkc-1									■		■														2
aex-6 dgk-1 egl-3 egl-30 egl-30gf egl-8 pkc-1 rab-3 sad-1 syd-2 unc-10 unc-2 unc-31 unc-36 wwp-1																				■				■	2
egl-3 egl-30 egl-30gf egl-8 pkc-1 rab-3 unc-10 unc-13 unc-18 unc-2 unc-31 unc-36 wwp-1										■				■											2
aex-6 dgk-1 egl-3 egl-30 egl-30gf egl-8 pkc-1 rab-3 unc-10 unc-13 unc-18 unc-2 unc-31 unc-36 wwp-1																	■	■							2
snb-1 unc-11 unc-13 unc-18																			■				■		2
snb-1 unc-11 unc-13 unc-18 unc-26 unc-57																			■				■		2
aex-3 egl-3 egl-30 egl-30gf egl-8 pkc-1 rab-3 unc-10 unc-13 unc-18 unc-2 unc-31 unc-36											■		■												2
egl-30gf rab-3 unc-13 unc-18 unc-2 unc-31				■				■																	2
aex-6 dgk-1 egl-3 egl-30 egl-30gf egl-8 pkc-1 unc-2 unc-31 unc-36 wwp-1																		■						■	2
egl-30gf rab-3 unc-2																	■				■				2
aex-3 aex-6 dgk-1 egl-10 egl-3 egl-30 egl-30gf egl-8 goa-1 pkc-1 rab-3 sad-1 syd-2 tomo-1 unc-10 unc-2 unc-31 unc-36 wwp-1																			■				■		2
aex-3 aex-6 dgk-1 egl-3 egl-30 egl-30gf egl-8 goa-1 pkc-1 rab-3 sad-1 syd-2 tomo-1 unc-10 unc-2 unc-31 unc-36 wwp-1																					■	■			2
aex-3 aex-6 dgk-1 egl-3 egl-30 egl-30gf egl-8 goa-1 pkc-1 rab-3 sad-1 snb-1 syd-2 tomo-1 unc-10 unc-13 unc-18 unc-2 unc-26 unc-31 unc-36 unc-57 wwp-1																		■		■					2
aex-3 egl-3 egl-30 egl-30gf egl-8 pkc-1 rab-3 unc-13 unc-18 unc-2 unc-36												■				■									2
aex-3 aex-6 dgk-1 egl-3 egl-30 egl-30gf egl-8 pkc-1 rab-3 sad-1 syd-2 unc-10 unc-2 unc-31 unc-36 wwp-1																				■				■	2
dgk-1 egl-10 sad-1 syd-2										■				■											2
egl-3 wwp-1			■																■						2
goa-1 sad-1 syd-2 wwp-1				■				■																	2
egl-3 egl-30 egl-30gf egl-8 pkc-1 rab-3 unc-10 unc-13 unc-18 unc-2 wwp-1										■				■											2
aex-6 egl-30gf rab-3 unc-13 unc-18 unc-2 unc-31 unc-36	■	■																							2
pkc-1 wwp-1																					■		■		2
aex-6 egl-3 egl-30 egl-30gf egl-8 pkc-1 rab-3 snb-1 unc-10 unc-11 unc-13 unc-18 unc-2 unc-31 unc-36		■				■																			2
dgk-1 goa-1 sad-1 syd-2 tomo-1			■				■																		2
aex-6 dgk-1 egl-10 egl-3 egl-30 egl-30gf egl-8 goa-1 pkc-1 rab-3 sad-1 syd-2 tomo-1 unc-10 unc-13 unc-18 unc-2 unc-31 unc-36 wwp-1				■				■																	2
dgk-1 goa-1 sad-1 syd-2 wwp-1				■				■																	2
aex-3 aex-6 egl-3 egl-30 egl-30gf egl-8 pkc-1 rab-3 snb-1 unc-10 unc-11 unc-13 unc-18 unc-2 unc-31 unc-36									■				■												2
aex-3 dgk-1 egl-10 egl-3 egl-30 egl-30gf egl-8 goa-1 pkc-1 rab-3 sad-1 syd-2 tomo-1 unc-10 unc-13 unc-18 unc-2 unc-31 unc-36 wwp-1											■					■									2
aex-6 dgk-1 egl-3 egl-30 egl-30gf egl-8 pkc-1 unc-10 unc-2 unc-31 unc-36 wwp-1																			■				■		2
aex-6 egl-3 egl-30 egl-30gf egl-8 pkc-1 rab-3 snb-1 unc-10 unc-11 unc-13 unc-18 unc-2 unc-31 unc-36 wwp-1										■				■											2
aex-3 aex-6 dgk-1 egl-10 egl-3 egl-30 egl-30gf egl-8 goa-1 pkc-1 rab-3 sad-1 snb-1 syd-2 tomo-1 unc-10 unc-13 unc-18 unc-2 unc-26 unc-31 unc-36 unc-57 wwp-1																		■		■					2
egl-30 egl-8 pkc-1 unc-36 wwp-1																						■		■	2
egl-30 egl-8 pkc-1 wwp-1																					■			■	2
egl-30 rab-3									■		■														2
egl-30 rab-3 unc-13 unc-18 unc-2 unc-31 unc-36											■														1
egl-30gf pkc-1															■										1
egl-30gf pkc-1 unc-10							■																		1
aex-3 dgk-1 egl-10 egl-3 egl-30 egl-30gf egl-8 goa-1 pkc-1 rab-3 sad-1 syd-2 unc-13 unc-18 unc-2 unc-31 unc-36 wwp-1																■									1
aex-6 dgk-1 egl-3 egl-30 egl-30gf egl-8 pkc-1 sad-1 syd-2 unc-2 unc-31 unc-36 wwp-1																				■					1
egl-30gf rab-3 unc-13 unc-18 unc-2 unc-31 unc-36													■												1
egl-30gf unc-10							■																		1
aex-3 dgk-1 egl-10 egl-3 egl-30 egl-30gf egl-8 goa-1 pkc-1 rab-3 sad-1 syd-2 unc-13 unc-18 unc-2 unc-36 wwp-1												■													1
egl-30gf unc-10 unc-13 unc-18 unc-2									■																1
egl-30gf unc-13 unc-18 unc-2 unc-31								■																	1
egl-3 egl-30 egl-30gf egl-8 pkc-1															■										1
aex-3 dgk-1 egl-10 egl-3 egl-30 egl-8 goa-1 rab-3 sad-1 syd-2 tomo-1 wwp-1							■																		1
aex-6 dgk-1 egl-3 egl-30 egl-8 pkc-1 sad-1 syd-2 unc-31 unc-36 wwp-1																				■					1
egl-3 egl-30 egl-30gf egl-8 pkc-1 rab-3 unc-10 unc-13 unc-18 unc-2 unc-31 unc-36								■																	1

Genes in Cluster	Clustering Method: Pearson's Correlation (uncentered)				Pearson's Correlation (centered)				Spearman's Rank Correlation				Kendall's Tau				Euclidean Distance				City Block Distance				score
	a	c	m	s	a	c	m	s	a	c	m	s	a	c	m	s	a	c	m	s	a	c	m	s	
aex-6 dgk-1 egl-3 egl-30 egl-8 pkc-1 unc-10 unc-31 unc-36 wwp-1																	■								1
egl-3 egl-30 egl-30gf egl-8 pkc-1 rab-3 unc-13 unc-18 unc-2												■													1
egl-3 egl-30 egl-30gf egl-8 pkc-1 rab-3 unc-13 unc-18 unc-2 unc-31								■																	1
aex-6 egl-30 egl-30gf egl-8 pkc-1 rab-3 unc-10 unc-13 unc-18 unc-2 unc-31 unc-36			■																						1
egl-3 egl-30 egl-30gf egl-8 rab-3 unc-13 unc-18 unc-2																■									1
aex-6 egl-30 egl-8 pkc-1 unc-10 unc-31 unc-36			■																						1
egl-3 egl-30 egl-8 pkc-1 unc-10	■																								1
aex-6 egl-30gf rab-3 unc-10 unc-13 unc-18 unc-2						■																			1
egl-3 egl-30 egl-8 pkc-1 unc-31 unc-36																				■					1
aex-3 aex-6 dgk-1 egl-10 egl-3 egl-30 egl-30gf egl-8 goa-1 pkc-1 rab-3 sad-1 snb-1 syd-2 unc-10 unc-11 unc-13 unc-18 unc-2 unc-31 unc-36 wwp-1												■													1
aex-3 aex-6 dgk-1 egl-10 egl-3 egl-30 egl-30gf egl-8 goa-1 pkc-1 rab-3 sad-1 syd-2 unc-11 unc-13 unc-18 unc-2 unc-36 wwp-1												■													1
aex-3 aex-6 dgk-1 egl-3 egl-30 egl-30gf egl-8 goa-1 pkc-1 rab-3 sad-1 syd-2 unc-10 unc-13 unc-18 unc-2 unc-31 unc-36 wwp-1																				■					1
aex-6 egl-30gf rab-3 unc-10 unc-13 unc-18 unc-2 unc-31						■																			1
egl-3 egl-30 egl-8 rab-3												■													1
aex-3 dgk-1 egl-10 goa-1 sad-1 syd-2 unc-10															■										1
aex-6 egl-30gf snb-1 unc-10 unc-11 unc-13 unc-18 unc-2									■																1
aex-6 egl-30gf unc-10 unc-2 unc-31 unc-36																							■		1
aex-3 aex-6 dgk-1 egl-3 egl-30 egl-30gf egl-8 pkc-1 rab-3 sad-1 syd-2 tomo-1 unc-10 unc-13 unc-18 unc-2 unc-26 unc-31 unc-36 unc-57 wwp-1																								■	1
aex-3 egl-10			■																						1
aex-6 egl-30gf unc-2 unc-31 unc-36																			■						1
egl-30 rab-3 unc-13 unc-18 unc-2											■														1
aex-3 dgk-1 egl-10 egl-3 egl-30 egl-30gf egl-8 goa-1 pkc-1 rab-3 sad-1 syd-2 unc-10 unc-13 unc-18 unc-2 unc-31 unc-36															■										1
rab-3 unc-13 unc-18 unc-2 unc-31 unc-36															■										1
aex-3 egl-30 rab-3									■																1
sad-1 syd-2 wwp-1				■																					1
aex-3 aex-6 dgk-1 egl-3 egl-30 egl-30gf egl-8 pkc-1 rab-3 sad-1 syd-2 tomo-1 unc-10 unc-13 unc-18 unc-2 unc-31 unc-36 wwp-1																								■	1
aex-3 egl-30gf unc-10											■														1
aex-6 egl-3 egl-30 egl-30gf egl-8 pkc-1 rab-3 unc-10 unc-13 unc-18 unc-2 unc-31						■																			1
aex-3 aex-6 dgk-1 egl-3 egl-30 egl-30gf egl-8 goa-1 pkc-1 rab-3 sad-1 syd-2 tomo-1 unc-10 unc-2 unc-26 unc-31 unc-36 unc-57 wwp-1																						■			1
aex-6 egl-3 egl-30 egl-8 pkc-1 unc-31 unc-36 wwp-1																				■					1
aex-6 snb-1 tomo-1 unc-11 unc-26 unc-57 wwp-1															■										1
aex-6 dgk-1 egl-3 egl-30 egl-8 pkc-1 rab-3 unc-31 unc-36 wwp-1																						■			1
aex-3 aex-6 dgk-1 egl-3 egl-30 egl-30gf egl-8 pkc-1 rab-3 sad-1 syd-2 tomo-1 unc-10 unc-2 unc-31 unc-36 wwp-1																								■	1
aex-3 egl-3 egl-30 egl-30gf egl-8 pkc-1 rab-3 unc-13 unc-18 unc-2												■													1
aex-6 snb-1 tomo-1 unc-11 wwp-1															■										1
aex-6 snb-1 unc-11 unc-26 unc-57											■														1
aex-3 aex-6 dgk-1 egl-10 egl-3 egl-30 egl-30gf egl-8 goa-1 pkc-1 rab-3 sad-1 snb-1 syd-2 unc-11 unc-13 unc-18 unc-2 unc-31 unc-36 wwp-1												■													1
aex-3 egl-3 egl-30 egl-30gf egl-8 pkc-1 rab-3 unc-13 unc-18 unc-2 unc-31 unc-36																■									1
aex-6 unc-10 unc-31 unc-36																							■		1
aex-3 aex-6 dgk-1 egl-3 egl-30 egl-30gf egl-8 pkc-1 rab-3 sad-1 syd-2 unc-10 unc-13 unc-18 unc-2 unc-31 unc-36 wwp-1																				■					1
aex-6 unc-13 unc-18 unc-2 unc-31 unc-36							■																		1
aex-3 dgk-1 egl-10 egl-3 goa-1 sad-1 syd-2 tomo-1 wwp-1			■																						1
aex-3 aex-6 dgk-1 egl-3 egl-30 egl-30gf egl-8 goa-1 pkc-1 rab-3 sad-1 syd-2 unc-10 unc-13 unc-18 unc-2 unc-26 unc-31 unc-36 unc-57 wwp-1																				■					1
egl-3 egl-30 egl-8 pkc-1 rab-3												■													1
aex-6 egl-30gf pkc-1 unc-10 unc-13 unc-18 unc-2 unc-31 unc-36							■																		1
aex-3 egl-3 egl-30 egl-30gf egl-8 rab-3 unc-13 unc-18 unc-2																■									1
dgk-1 egl-10 egl-3 egl-30 egl-30gf egl-8 goa-1 pkc-1 rab-3 sad-1 syd-2 tomo-1 unc-10 unc-13 unc-18 unc-2 unc-31 unc-36 wwp-1				■																					1
dgk-1 egl-10 egl-3 egl-30 egl-30gf egl-8 goa-1 pkc-1 rab-3 sad-1 syd-2 tomo-1 unc-13 unc-18 unc-2 unc-31 unc-36 wwp-1				■																					1
aex-3 egl-3 egl-30 egl-30gf egl-8 rab-3 unc-13 unc-18 unc-2 unc-36																■									1
dgk-1 egl-10 egl-3 egl-30 egl-8 goa-1 pkc-1 sad-1 syd-2 tomo-1 unc-36 wwp-1				■																					1
aex-3 egl-3 egl-30 egl-8 pkc-1 rab-3									■																1
dgk-1 egl-10 egl-3 egl-30 egl-8 goa-1 pkc-1 sad-1 syd-2 tomo-1 wwp-1				■																					1
aex-3 aex-6 dgk-1 egl-3 egl-30 egl-30gf egl-8 goa-1 pkc-1 rab-3 sad-1 snb-1 syd-2 tomo-1 unc-10 unc-13 unc-18 unc-2 unc-31 unc-36 wwp-1																		■							1
aex-3 aex-6 dgk-1 egl-3 egl-30 egl-30gf egl-8 pkc-1 rab-3 snb-1 unc-10 unc-11 unc-13 unc-18 unc-2 unc-26 unc-31 unc-36 unc-57 wwp-1																	■								1
aex-3 egl-3 egl-30 egl-8 pkc-1 rab-3 unc-31 unc-36									■																1
dgk-1 egl-10 goa-1 sad-1 syd-2 tomo-1							■																		1
aex-3 egl-3 egl-30 egl-8 rab-3 wwp-1							■																		1
dgk-1 egl-3 egl-30 egl-30gf egl-8 pkc-1 unc-2 unc-31 unc-36 wwp-1																		■							1
aex-3 aex-6 dgk-1 egl-3 egl-30 egl-30gf egl-8 pkc-1 rab-3 unc-10 unc-13 unc-18 unc-2 unc-26 unc-31 unc-36 unc-57 wwp-1																	■								1
aex-3 egl-3 egl-30gf egl-8 pkc-1 unc-10											■														1
dgk-1 egl-3 egl-30 egl-8 pkc-1 unc-10 wwp-1																			■						1
dgk-1 egl-3 egl-30 egl-8 pkc-1 unc-36 wwp-1																						■			1
aex-3 aex-6 dgk-1 egl-10 egl-3 egl-30 egl-30gf egl-8 goa-1 pkc-1 rab-3 sad-1 snb-1 syd-2 unc-11 unc-13 unc-18 unc-2 unc-36 wwp-1												■													1
aex-3 aex-6 dgk-1 egl-3 egl-30 egl-30gf egl-8 goa-1 pkc-1 rab-3 sad-1 snb-1 syd-2 unc-10 unc-13 unc-18 unc-2 unc-26 unc-31 unc-36 unc-57 wwp-1																				■					1
aex-3 aex-6 dgk-1 egl-3 egl-30 egl-30gf egl-8 pkc-1 rab-3 unc-10 unc-13 unc-18 unc-2 unc-31 unc-36 wwp-1																	■								1
dgk-1 egl-3 egl-30 egl-8 pkc-1 wwp-1																							■		1
dgk-1 goa-1											■														1
aex-6 dgk-1 egl-10 egl-3 egl-30 egl-30gf egl-8 goa-1 pkc-1 rab-3 sad-1 snb-1 syd-2 tomo-1 unc-10 unc-11 unc-13 unc-18 unc-2 unc-31 unc-36 wwp-1				■																					1
aex-6 dgk-1 egl-3 egl-30 egl-30gf egl-8 goa-1 pkc-1 rab-3 sad-1 snb-1 syd-2 tomo-1 unc-10 unc-13 unc-18 unc-2 unc-31 unc-36 wwp-1																		■							1
dgk-1 pkc-1 wwp-1																							■		1
aex-6 dgk-1 egl-3 egl-30 egl-30gf egl-8 goa-1 pkc-1 rab-3 sad-1 syd-2 tomo-1 unc-10 unc-13 unc-18 unc-2 unc-31 unc-36 wwp-1																		■							1
dgk-1 unc-10																			■						1
aex-6 dgk-1 egl-3 egl-30 egl-30gf egl-8 goa-1 pkc-1 rab-3 sad-1 syd-2 tomo-1 unc-10 unc-2 unc-31 unc-36 wwp-1																						■			1
aex-6 dgk-1 egl-3 egl-30 egl-30gf egl-8 goa-1 pkc-1 rab-3 sad-1 syd-2 unc-10 unc-2 unc-31 unc-36 wwp-1																						■			1
egl-30 egl-8 pkc-1 unc-10			■																						1
egl-30 egl-8 pkc-1 unc-36																						■			1
aex-3 aex-6 dgk-1 egl-3 egl-30 egl-30gf egl-8 goa-1 pkc-1 rab-3 sad-1 syd-2 tomo-1 unc-10 unc-13 unc-18 unc-2 unc-31 unc-36 wwp-1																					■				1
aex-3 dgk-1 egl-10 egl-3 egl-30 egl-30gf egl-8 goa-1 pkc-1 rab-3 sad-1 syd-2 tomo-1 unc-13 unc-18 unc-2 unc-31 unc-36 wwp-1																■									1
aex-6 dgk-1 egl-3 egl-30 egl-30gf egl-8 pkc-1 rab-3 sad-1 syd-2 unc-2 unc-31 unc-36 wwp-1																				■					1

Linkage method: a = average, c = centroid, s = single, m = complete.
