# Supplementary material for: Profiling Synaptic Proteins Identifies Regulators of Insulin Secretion and Lifespan
Source: PLoS Genet. 2008 Nov 28;4(11):e1000283. doi: 10.1371/journal.pgen.1000283 (PMC2582949; doi:10.1371/journal.pgen.1000283)
Supplement: Table S2 — Quantitative imaging of presynaptic markers. (0.77 MB PDF) [file pgen.1000283.s005.pdf]

Table S2 - Quantitative imaging of presynaptic markers

Percent difference from wild type controls

Marker	Parameter	Mutant																								
		unc-13	*unc-18*	*unc-2*	*unc-10*	*unc-57*	*dgk-1*	*goa-1*	*egl-30*	*egl-30gf*	*egl-3*	*egl-8*	*pkc-1*	*snb-1*	*unc-31*	*unc-36*	*sad-1*	*syd-2*	*unc-11*	*unc-26*	*tomo-1*	*aex-3*	*aex-6*	*rab-3*	*egl-10*	*wwp-1*
GFP::SNB-1	Punctal Fluorescence	43%	59%	44%	9%	-30%	-9%	-17%	4%	27%	-21%	-7%	-7%		23%	12%	-38%	-24%	25%	-2%	5%	8%	38%	22%	-41%	-31%
	Inter-punctal Fluorescence	-15%	-19%	18%	-16%	15%	-2%	-5%	-14%	33%	-2%	2%	-9%		-13%	25%	-25%	-15%	136%	10%	42%	-26%	16%	-18%	-39%	-7%
	Full Width Half Max	-2%	-5%	6%	11%	-11%	4%	-1%	-7%	0%	1%	-3%	-6%		-4%	-5%	0%	-7%	22%	-21%	-13%	5%	-1%	3%	-2%	-7%
	Inter-punctal Distance	-8%	-6%	-1%	6%	13%	2%	3%	-3%	4%	6%	12%	0%		-6%	0%	17%	-1%	19%	-9%	-3%	3%	-8%	-1%	0%	1%
GFP::SYD-2	Punctal Fluorescence	3%	7%	0%	4%	5%	-1%	14%	8%	27%	1%	7%	5%	-5%	-5%	-7%	11%		-2%	3%	12%	13%	11%	23%	-18%	7%
	Inter-punctal Distance	-2%	1%	10%	-1%	-8%	-2%	1%	3%	10%	-1%	7%	-6%	5%	2%	10%	9%		1%	-4%	-2%	5%	7%	6%	7%	6%
SNN-1::Venus	Punctal Fluorescence	-32%	-48%	-12%	-20%	7%	-43%	-13%	-5%	-16%	-2%	-3%	-23%	-18%	16%	2%	-20%	-35%	-10%	37%	8%	2%	-6%		10%	-6%
	Inter-punctal Fluorescence	-26%	-37%	-15%	-24%	-6%	-29%	3%	-10%	-12%	-15%	-22%	-1%	6%	0%	11%	-5%	24%	37%	13%	15%	10%	8%		-12%	15%
	Inter-punctal Distance	-14%	-13%	-1%	-11%	-9%	3%	3%	-2%	-1%	0%	-9%	-5%	-9%	-12%	-7%	6%	23%	-19%	-12%	-3%	-9%	-13%		17%	-1%
UNC-10::GFP	Punctal Fluorescence	20%	55%	17%		4%	-8%	23%	-7%	-11%	33%	10%	-15%	7%	-1%	4%	-23%	-19%	27%	35%	-13%	-26%	8%	-8%	-33%	-9%
	Inter-punctal Distance	6%	6%	7%		-3%	3%	8%	2%	18%	3%	6%	5%	12%	8%	7%	22%	13%	-6%	-9%	7%	11%	-3%	6%	23%	3%
Venus::RAB-3	Punctal Fluorescence	26%	62%	16%	1%	-3%	-11%	-12%	-35%	-15%	-21%	-16%	-5%	61%	-1%	4%	0%	11%	82%	-11%	-6%	-81%	11%		-44%	-2%
	Inter-punctal Fluorescence	-41%	-19%	-13%	-26%	-17%	-9%	-17%	-11%	-20%	6%	1%	-5%	-44%	-27%	5%	-3%	-3%	-30%	-11%	11%	-55%	3%		-42%	-10%
	Full Width Half Max	-5%	-9%	-11%	-13%	-6%	1%	-1%	-3%	-11%	-2%	7%	2%	-13%	-1%	0%	6%	-6%	-12%	1%	5%	-2%	-2%		-5%	2%
	Inter-punctal Distance	-2%	-8%	-5%	-8%	0%	-1%	-1%	1%	-1%	4%	-2%	-4%	5%	-4%	0%	6%	2%	-13%	2%	-1%	57%	-3%		3%	-1%
Gelsolin::Venus	Punctal Fluorescence	-32%	6%	-2%	-8%	30%	-10%	37%	-24%	26%	-9%	1%	-6%	33%	-16%	-7%	-15%	-10%	46%	11%	-31%	-9%	-4%	-9%	-33%	-11%
	Inter-punctal Fluorescence	-32%	16%	4%	14%	39%	-10%	7%	-10%	-6%	15%	-9%	-14%	36%	-24%	-11%	-3%	-3%	48%	56%	2%	-12%	19%	-4%	-15%	-13%
	Inter-punctal Distance	17%	12%	-1%	2%	1%	2%	5%	-3%	-2%	1%	0%	-4%	11%	7%	4%	3%	9%	16%	11%	20%	7%	1%	1%	8%	1%
APT-4::GFP	Punctal Fluorescence	-21%	-25%	-26%	-28%	21%		12%	-21%	-18%	-27%	-12%	0%	-19%	-6%	-13%	-1%	-14%	-1%	20%	-4%		-6%	-5%	-22%	-7%
	Full Width Half Max	39%	24%	30%	10%	7%		10%	-4%	13%	4%	-1%	-7%	46%	8%	-12%	2%	20%	44%	-2%	5%		27%	33%	5%	-7%
	Inter-punctal Distance	6%	0%	-1%	5%	-10%		4%	-1%	2%	2%	-6%	1%	11%	2%	-3%	10%	12%	-4%	-9%	-1%		5%	-7%	17%	1%
INS-22::Venus	Punctal Fluorescence	32%	28%	13%	2%	-14%	-21%	-36%	9%	3%	15%	18%	19%	-39%	18%	11%	-43%	-38%	-1%		-58%	-19%	-2%	21%	-26%	-11%
	Inter-punctal Fluorescence	4%	6%	30%	-5%	-8%	-13%	-8%	36%	20%	15%	37%	10%	-23%	15%	-3%	-10%	-14%	10%		-15%	-17%	9%	23%	9%	11%
	Full Width Half Max	23%	30%	4%	6%	4%	-4%	-24%	0%	7%	3%	8%	8%	-4%	6%	4%	-11%	-13%	11%		-25%	10%	0%	7%	-14%	0%
	Inter-punctal Distance	13%	21%	7%	3%	7%	3%	10%	5%	8%	8%	1%	1%	8%	2%	2%	0%	5%	1%		7%	8%	2%	2%	4%	0%
ITSN-1::GFP	Punctal Fluorescence	3%	2%	2%	-33%	27%	-11%	-34%	-28%	-8%	-19%	-24%	-18%	-3%	-13%	-16%	-30%	-40%	-8%	31%	-17%	-27%	-26%	-14%	-6%	-18%
	Inter-punctal Fluorescence	-23%	-10%	-11%	-21%	-15%	-13%	-22%	-32%	-20%	-27%	-30%	-22%	2%	-18%	-26%	-25%	-15%	-11%	20%	-27%	-25%	-30%	-1%	11%	-16%
	Full Width Half Max	-17%	-7%	-9%	7%	-13%	-3%	20%	-7%	1%	-11%	-2%	0%	-4%	3%	-3%	-2%	2%	-16%	-11%	-4%	-1%	-4%	-4%	10%	-6%
	Inter-punctal Distance	-3%	0%	-5%	7%	-6%	-2%	9%	-1%	1%	-3%	-1%	1%	3%	2%	0%	4%	7%	-5%	-9%	-2%	-2%	-2%	-5%	16%	-4%

significant increase relative to wild type (p<0.05)   significant decrease relative to wild type (p<0.05)

Student's T-Test *p*-value between mutant and wild type controls

Marker	Parameter	Mutant																								
		unc-13	*unc-18*	*unc-2*	*unc-10*	*unc-57*	*dgk-1*	*goa-1*	*egl-30*	*egl-30gf*	*egl-3*	*egl-8*	*pkc-1*	*snb-1*	*unc-31*	*unc-36*	*sad-1*	*syd-2*	*unc-11*	*unc-26*	*tom-1*	*aex-3*	*aex-6*	*rab-3*	*egl-10*	*wwp-1*
GFP::SNB-1	Punctal Fluorescence	1.9E-02	6.3E-14	1.4E-08	1.2E-01	1.2E-07	2.1E-01	6.0E-04	6.4E-01	5.3E-06	1.4E-05	3.3E-01	2.2E-01		1.0E-04	1.4E-01	1.7E-11	7.6E-04	2.3E-04	3.1E-01	5.0E-01	7.3E-02	6.4E-10	1.6E-03	3.3E-09	2.4E-09
	Inter-punctal Fluorescence	1.4E-01	9.2E-04	1.4E-03	9.5E-04	3.1E-02	7.0E-01	3.4E-01	2.2E-02	2.2E-09	7.9E-01	8.6E-01	2.2E-01		1.1E-01	9.0E-03	7.1E-04	1.0E-01	7.7E-13	2.5E-02	1.2E-04	1.1E-06	5.9E-02	5.3E-03	9.7E-11	2.0E-01
	Full Width Half Max	1.1E-01	2.2E-01	2.9E-01	4.3E-03	1.8E-03	2.2E-01	5.2E-01	1.8E-02	8.8E-01	8.4E-01	1.9E-01	1.2E-01		1.0E-01	1.7E-01	6.9E-01	9.8E-02	4.9E-05	2.0E-07	1.7E-02	9.1E-02	7.1E-01	3.6E-01	8.0E-01	6.1E-02
	Inter-punctal Distance	8.9E-01	2.1E-02	6.6E-01	8.6E-02	1.8E-02	9.6E-01	2.2E-01	4.2E-01	8.0E-02	3.4E-02	1.0E-01	6.9E-01		2.2E-02	8.3E-01	7.6E-05	6.4E-01	2.9E-04	4.5E-03	7.9E-01	4.0E-01	1.1E-04	7.4E-01	3.3E-01	4.3E-01
GFP::SYD-2	Punctal Fluorescence	6.1E-01	2.0E-01	5.2E-01	1.6E-01	5.4E-01	5.1E-01	7.6E-04	1.5E-01	1.2E-08	5.5E-01	1.4E-01	3.0E-01	1.7E-01	3.3E-01	2.6E-01	1.8E-01		9.4E-01	3.7E-01	7.4E-04	1.2E-03	4.0E-02	1.7E-08	1.9E-04	2.2E-02
	Inter-punctal Distance	1.2E-01	6.6E-01	2.6E-02	6.4E-01	2.6E-03	5.4E-01	9.7E-01	5.7E-01	1.9E-02	9.7E-01	1.7E-01	3.5E-02	5.3E-02	5.1E-01	9.2E-03	3.1E-03		4.6E-01	2.1E-01	4.8E-01	3.6E-02	3.7E-02	4.9E-03	9.1E-03	3.6E-02
SNN-1::Venus	Punctal Fluorescence	1.3E-08	1.5E-15	2.0E-01	6.4E-04	6.6E-01	4.6E-11	1.5E-01	1.9E-01	6.1E-03	8.6E-01	6.6E-01	1.5E-03	2.1E-01	4.8E-02	9.8E-01	4.1E-03	1.0E-06	1.1E-01	2.9E-05	1.8E-01	4.3E-01	5.8E-02		1.3E-01	4.1E-01
	Inter-punctal Fluorescence	7.6E-05	6.4E-10	2.8E-02	1.9E-04	3.6E-01	6.3E-06	2.1E-01	3.2E-01	6.0E-02	9.4E-02	2.7E-03	8.8E-01	3.0E-01	9.4E-01	1.3E-02	2.2E-01	3.0E-05	6.1E-11	1.9E-03	8.7E-03	1.9E-02	3.7E-02		5.2E-04	4.2E-04
	Inter-punctal Distance	3.3E-10	1.3E-06	8.2E-01	2.0E-05	1.7E-03	3.0E-01	1.4E-01	5.3E-01	8.2E-01	9.6E-01	8.0E-03	3.5E-01	3.1E-03	1.7E-05	1.6E-03	3.7E-02	6.2E-03	1.8E-07	3.1E-05	2.7E-01	1.2E-02	3.8E-07		2.0E-04	6.5E-01
UNC-10::GFP	Punctal Fluorescence	4.6E-02	9.1E-04	2.0E-01		5.5E-01	1.1E-01	1.5E-02	2.0E-01	6.6E-02	2.0E-02	2.7E-01	2.1E-01	3.0E-01	7.4E-01	4.9E-01	2.3E-02	1.1E-02	5.9E-02	2.2E-03	1.1E-01	5.5E-04	3.5E-01	5.0E-01	1.6E-04	3.3E-01
	Inter-punctal Distance	1.9E-02	4.9E-03	8.5E-02		6.1E-01	7.7E-02	6.8E-03	2.1E-01	4.0E-05	2.4E-01	7.6E-02	9.6E-02	1.1E-04	3.0E-02	2.1E-01	3.2E-06	3.2E-03	4.4E-02	3.2E-05	3.9E-02	1.3E-04	2.6E-01	3.5E-02	5.4E-06	3.3E-01
Venus::RAB-3	Punctal Fluorescence	2.0E-03	2.6E-03	1.6E-01	4.9E-01	9.8E-01	4.4E-01	3.2E-01	3.1E-05	1.6E-01	5.1E-02	8.7E-02	8.6E-01	2.0E-06	9.1E-01	4.3E-01	9.1E-01	1.9E-01	1.9E-09	4.4E-01	6.0E-01	2.8E-25	8.6E-02		2.0E-05	5.1E-01
	Inter-punctal Fluorescence	2.4E-09	1.1E-01	1.2E-01	1.8E-05	1.5E-03	1.7E-01	1.5E-02	1.2E-01	2.4E-04	6.8E-01	9.0E-01	4.9E-01	9.1E-11	3.2E-06	5.8E-01	7.3E-01	6.8E-01	2.4E-04	4.7E-02	1.8E-01	1.4E-22	6.9E-01		2.3E-12	6.1E-02
	Full Width Half Max	5.3E-02	3.5E-03	1.5E-03	3.2E-04	1.5E-01	9.3E-01	9.5E-01	5.8E-01	4.9E-03	8.4E-01	1.1E-01	7.9E-01	9.7E-05	6.1E-01	9.1E-01	1.8E-01	2.5E-01	5.0E-06	6.9E-01	3.0E-01	1.1E-01	3.1E-01		6.3E-01	6.3E-01
	Inter-punctal Distance	1.2E-01	2.1E-04	5.8E-01	2.1E-02	6.7E-01	5.2E-01	4.2E-01	5.6E-01	4.0E-01	2.1E-01	6.4E-01	7.4E-02	1.7E-01	1.4E-01	5.3E-01	1.0E-01	4.9E-01	4.4E-06	3.7E-01	8.6E-01	7.4E-06	7.1E-01		9.1E-02	7.4E-01
Gelsolin::Venus	Punctal Fluorescence	3.5E-05	2.3E-01	9.7E-01	6.6E-01	1.3E-03	2.1E-01	9.5E-07	5.2E-03	2.8E-03	5.8E-01	9.8E-01	2.4E-01	1.4E-05	1.2E-02	1.7E-01	2.0E-02	1.3E-01	4.1E-04	4.9E-02	4.0E-05	1.6E-01	6.2E-01	1.6E-01	2.3E-06	1.4E-01
	Inter-punctal Fluorescence	1.6E-05	1.4E-01	6.9E-01	2.0E-01	3.1E-04	2.2E-01	4.2E-01	2.4E-01	4.8E-01	1.8E-01	4.0E-01	8.2E-02	2.6E-03	1.3E-04	2.1E-01	7.1E-01	7.5E-01	1.2E-03	8.8E-09	8.6E-01	1.3E-01	4.4E-02	6.0E-01	1.0E-01	1.2E-01
	Inter-punctal Distance	2.2E-04	3.1E-03	8.9E-01	4.6E-01	7.3E-01	7.4E-01	2.7E-01	6.9E-01	1.4E-01	2.9E-01	6.7E-01	1.8E-01	1.9E-03	1.2E-01	2.8E-01	3.0E-01	4.7E-02	2.9E-05	1.8E-03	1.2E-02	2.7E-02	7.3E-01	4.4E-01	4.5E-02	7.6E-01
APT-4::GFP	Punctal Fluorescence	2.2E-07	9.4E-07	7.4E-07	1.4E-08	5.4E-03		6.2E-02	1.2E-03	1.3E-03	1.4E-07	9.3E-03	9.6E-01	5.5E-04	7.1E-01	5.5E-02	6.3E-01	6.9E-02	8.1E-01	5.5E-06	4.7E-01		5.2E-01	2.8E-01	4.7E-06	1.6E-01
	Full Width Half Max	1.3E-11	4.8E-06	7.5E-05	2.5E-02	8.8E-02		1.7E-01	1.9E-01	9.3E-03	4.5E-01	9.1E-01	2.0E-01	2.5E-14	3.0E-01	5.6E-04	9.6E-01	6.1E-05	1.1E-15	4.7E-01	2.8E-01		6.4E-05	4.2E-06	2.3E-01	1.3E-01
	Inter-punctal Distance	8.2E-03	4.4E-01	6.8E-01	5.9E-03	1.6E-05		1.0E-01	8.0E-01	3.0E-01	4.0E-01	5.6E-02	7.0E-01	1.0E-05	3.0E-01	1.6E-01	1.5E-03	4.4E-04	7.3E-02	1.8E-04	8.9E-01		7.0E-02	1.3E-03	8.2E-06	3.5E-01
INS-22::Venus	Punctal Fluorescence	4.7E-04	7.0E-07	1.8E-02	5.7E-01	1.1E-02	4.5E-05	1.2E-16	1.3E-01	2.6E-01	2.9E-01	3.7E-03	6.3E-04	4.1E-10	1.6E-03	1.3E-02	3.0E-24	2.1E-19	7.0E-01		9.1E-32	4.7E-04	5.3E-01	7.0E-05	3.4E-06	2.7E-02
	Inter-punctal Fluorescence	5.3E-01	3.6E-01	1.0E-05	4.0E-01	1.3E-01	1.6E-02	1.4E-01	5.4E-05	1.4E-02	2.9E-02	4.0E-05	8.1E-02	2.8E-03	1.2E-01	6.8E-01	3.7E-02	4.6E-02	1.5E-01		1.6E-02	4.5E-04	2.3E-01	3.3E-03	2.6E-01	1.1E-01
	Full Width Half Max	6.2E-10	2.9E-06	1.4E-01	8.5E-03	3.6E-02	1.2E-01	1.4E-19	9.4E-01	8.0E-03	2.8E-01	1.5E-02	2.1E-04	2.1E-01	3.8E-01	9.8E-02	2.3E-04	6.2E-06	4.8E-05		4.6E-16	4.3E-04	1.0E+00	4.4E-02	1.6E-06	5.5E-01
	Inter-punctal Distance	5.6E-03	4.7E-05	1.8E-02	1.9E-01	5.4E-03	1.9E-01	6.3E-04	4.5E-02	8.2E-04	2.9E-03	2.5E-01	5.7E-01	2.5E-02	9.1E-01	2.3E-01	4.9E-01	2.1E-01	6.1E-01		3.1E-02	1.2E-03	6.3E-01	3.1E-01	1.3E-01	3.5E-01
ITSN-1::GFP	Punctal Fluorescence	8.5E-01	3.6E-01	5.8E-01	1.9E-20	2.9E-09	2.3E-02	1.5E-04	1.7E-08	5.6E-02	1.2E-04	3.3E-06	6.2E-05	5.6E-01	1.7E-03	5.1E-03	2.7E-17	7.4E-17	1.5E-01	1.5E-10	7.1E-05	1.4E-08	2.1E-11	2.4E-03	9.3E-02	2.5E-04
	Inter-punctal Fluorescence	2.3E-05	5.7E-02	4.7E-02	3.2E-08	1.8E-04	1.5E-02	2.0E-03	5.2E-09	2.8E-04	5.8E-06	2.9E-13	7.1E-05	7.1E-01	3.2E-04	1.2E-04	2.4E-10	4.8E-03	9.8E-04	1.5E-02	4.7E-07	7.4E-10	1.3E-13	8.3E-01	2.5E-02	4.7E-03
	Full Width Half Max	7.9E-11	1.0E-02	2.4E-03	7.3E-02	3.6E-07	2.4E-01	1.3E-06	8.5E-03	6.0E-01	5.2E-05	6.3E-01	8.0E-01	6.5E-02	1.2E-01	5.1E-01	4.7E-01	2.6E-01	2.2E-08	4.7E-07	1.7E-01	8.2E-01	1.0E-01	1.1E-01	1.1E-02	1.5E-02
	Inter-punctal Distance	1.2E-01	8.7E-01	2.9E-02	8.3E-04	2.3E-05	1.9E-01	6.7E-04	9.6E-01	8.6E-01	2.2E-01	5.3E-01	9.1E-01	2.5E-01	1.5E-01	6.5E-01	4.4E-02	7.8E-04	5.7E-02	3.4E-04	3.2E-01	4.0E-01	4.3E-01	7.1E-02	2.8E-05	1.0E-01

Significance levels: $p < 0.05$ $p < 0.01$ $p < 0.001$
