# Supplementary material for: Profiling Synaptic Proteins Identifies Regulators of Insulin Secretion and Lifespan
Source: PLoS Genet. 2008 Nov 28;4(11):e1000283. doi: 10.1371/journal.pgen.1000283 (PMC2582949; doi:10.1371/journal.pgen.1000283)
Supplement: Table S3 — Scores for clustering. (0.05 MB PDF) [file pgen.1000283.s006.pdf]

Table S3 - Scores for clustering

	Marker																												
	GFP::SNB-1				SYD::GFP		SNN-1::Venus			UNC-10::GFP		Venus::RAB-3				Gelsolin::Venus			APT-4::GFP			INS-22::Venus				ITSN-1::GFP			
**Gene**	**PF**	**IPF**	**FWHM**	**IPD**	**PF**	**IPD**	**PF**	**IPF**	**IPD**	**PF**	**IPD**	**PF**	**IPF**	**FWHM**	**IPD**	**PF**	**IPF**	**IPD**	**PF**	**FWHM**	**IPD**	**PF**	**IPF**	**FWHM**	**IPD**	**PF**	**IPF**	**FWHM**	**IPD**
snb-1					-1.38	2.48	-1.75	1.37	-3.01	1.04	3.98	5.01	-5.74	-3.25	1.37	4.48	3.89	3.15	-3.53	8.45	4.58	-6.82	-3.06	-1.26	2.27	-0.58	0.37	-1.85	1.15
unc-13	7.36	-2.07	-0.69	-2.34	0.52	-1.21	-4.81	-3.40	-5.98	2.42	2.39	3.18	-6.57	-1.70	-1.36	-3.65	-3.28	3.86	-4.54	7.68	2.70	5.09	0.63	6.75	2.83	0.19	-4.33	-6.83	-1.56
unc-18	8.49	-3.40	-1.22	-2.33	1.29	-0.35	-6.79	-4.89	-5.08	4.59	2.89	3.73	-1.91	-2.99	-3.48	1.22	1.48	3.04	-3.83	4.89	0.77	5.26	0.91	8.29	6.44	0.91	-1.91	-2.59	-0.17
unc-2	6.07	3.26	1.07	0.58	-0.64	2.26	-1.28	-1.75	-0.23	1.29	2.00	1.43	-1.58	-3.26	-0.63	0.04	0.40	0.13	-5.29	5.15	0.42	3.09	4.63	1.97	3.01	0.55	-2.00	-3.07	-2.20
unc-31	4.00	-1.59	-1.63	-2.32	-0.98	0.67	2.00	0.07	-4.44	-0.33	2.21	-0.11	-3.57	-0.51	-1.49	-2.55	-2.86	1.55	-0.37	1.03	1.04	2.79	1.01	1.06	0.03	-3.17	-3.66	2.12	1.43
unc-36	2.03	2.65	-1.37	-0.21	-1.15	2.71	-0.03	2.51	-3.22	0.69	1.28	0.79	0.56	-0.11	-0.63	-1.38	-1.26	1.08	-1.93	-2.43	-1.41	2.52	-0.41	1.98	1.22	-2.83	-3.91	-0.65	-0.45
sad-1	-7.39	-3.47	0.40	4.09	1.74	3.02	-2.91	-1.22	2.11	-2.33	5.07	0.11	-0.34	1.36	1.64	-2.34	-0.38	1.35	-0.49	-0.05	3.23	-8.87	-1.56	-3.81	-0.69	-7.04	-5.04	-0.73	2.02
syd-2	-3.44	-1.64	-1.67	0.47			-5.09	5.75	5.33	-2.58	3.92	1.32	-0.41	-1.16	0.70	-1.53	-0.32	2.57	-1.83	4.13	5.19	-7.51	-2.01	-4.74	1.62	-9.05	-2.85	1.13	3.41
unc-10	1.56	-3.38	2.91	1.73	1.42	-0.38	-3.04	-3.26	-4.43			0.69	-3.86	-3.19	-2.36	-0.45	1.29	0.74	-5.53	2.28	2.81	0.57	-0.85	2.68	1.32	-7.91	-4.44	2.34	3.39
unc-11	3.78	13.75	6.25	5.85	-0.08	0.74	-1.60	7.09	-5.50	1.92	-2.05	6.54	-3.79	-3.26	-4.83	4.81	4.72	4.30	-0.24	8.98	-1.80	-0.46	1.46	4.22	0.51	-1.43	-2.12	-5.81	-1.92
unc-26	-1.02	2.25	-5.68	-2.96	0.90	-1.27	5.61	3.16	-4.29	4.10	-3.62	-0.78	-1.59	-0.31	0.91	1.99	6.08	3.18	4.71	-0.50	-3.84					6.72	3.32	-4.14	-3.64
unc-57	-5.62	2.18	-3.18	2.39	0.73	-2.54	0.40	-0.82	-3.20	0.60	-0.51	-0.03	-2.89	-1.45	-0.43	3.31	3.75	0.35	2.85	1.40	-4.55	-2.58	-1.51	2.12	2.83	6.18	-3.02	-5.24	-3.01
dgk-1	-1.25	-0.39	1.22	0.05	-0.66	-0.62	-5.86	-3.88	1.04	-1.61	1.79	-0.78	-1.39	0.08	-0.65	-1.25	-1.22	0.34				-4.24	-2.45	-1.55	1.32	-2.29	-2.44	-1.18	-1.32
goa-1	-3.10	-0.96	-0.64	1.24	3.47	0.04	-1.43	1.27	1.50	2.49	2.77	-0.99	-2.49	0.06	-0.81	5.11	0.81	1.10	2.20	1.38	1.66	-8.26	-1.47	-10.99	3.52	-6.11	-4.01	7.39	3.45
tomo-1	0.69	4.54	-2.45	0.27	3.46	-0.70	1.34	3.48	-1.10	-1.61	2.13	-0.53	1.34	1.05	-0.18	-4.23	0.17	4.85	-0.72	1.08	-0.13	-12.56	-2.44	-9.40	2.82	-4.05	-5.19	-1.39	-0.99
aex-3	1.81	-3.46	1.70	0.85	3.31	2.12	0.80	2.38	-2.53	-3.54	3.94	-7.57	-7.01	-1.61	9.81	-1.40	-1.51	2.22				-3.60	-2.69	3.63	3.32	-5.89	-5.08	-0.23	-0.85
aex-6	6.61	1.90	-0.37	-3.19	2.10	2.06	-1.42	2.11	-5.31	0.93	-1.13	1.73	0.40	-1.02	-0.37	-0.49	2.03	0.34	-0.64	4.12	1.82	-0.64	1.20	0.00	0.48	-6.10	-5.89	-1.65	-0.79
rab-3	3.21	-2.25	0.93	-0.34	6.01	2.86				-0.68	2.14					-1.42	-0.52	0.77	-0.83	6.20	-3.28	4.12	3.00	2.03	1.01	-3.07	-0.21	-1.63	-1.81
egl-10	-6.30	-4.80	0.26	0.97	-3.95	2.40	1.51	-3.54	5.58	-4.19	5.02	-4.45	-6.05	-0.61	2.36	-4.90	-1.65	2.98	-4.75	1.20	6.77	-4.88	1.12	-5.06	1.53	-1.69	2.26	3.40	7.37
egl-3	-4.52	-0.27	-0.20	2.14	-0.60	0.04	-0.18	-1.69	0.05	2.37	1.19	-1.98	0.55	-0.20	1.27	-0.56	1.37	1.43	-4.25	0.75	0.84	1.38	2.21	1.80	3.05	-3.92	-4.65	-4.13	-1.24
egl-30	0.46	-2.33	-2.39	-0.80	1.46	0.58	-1.31	-0.99	-0.63	-1.29	1.26	-3.63	-1.58	-0.55	0.76	-2.87	-1.00	-0.40	-3.36	-1.31	-0.25	1.54	5.27	0.09	2.02	-5.85	-6.08	-2.66	-0.05
egl-30gf	4.76	6.45	0.16	1.77	6.14	1.98	-2.17	-1.40	0.28	-1.86	5.46	-1.41	-3.63	-2.87	-0.85	3.07	-0.62	-1.29	-3.31	2.65	1.04	1.28	2.96	3.08	3.43	-1.92	-3.69	0.53	-0.18
egl-8	-0.98	0.18	-1.31	1.64	1.48	1.38	-0.44	-2.17	-2.70	1.12	1.80	-1.73	0.12	1.62	-0.47	0.03	-0.85	-0.42	-2.66	-0.09	-1.93	2.97	5.42	3.10	1.17	-4.77	-5.77	-0.49	-0.62
pkc-1	-1.23	-1.24	-1.58	0.40	1.05	-1.78	-2.84	-0.15	-0.94	-1.25	1.68	-0.17	-0.70	0.27	-1.81	-1.19	-1.62	-1.36	0.05	-1.28	0.39	3.51	1.76	3.82	0.57	-4.08	-4.05	0.25	0.11
wwp-1	-5.25	-0.97	-1.89	1.00	2.32	2.12	-0.82	3.61	-0.59	-0.98	1.36	-0.66	-1.34	0.49	-0.34	-1.48	-1.57	0.31	-1.43	-1.50	0.94	-2.23	1.62	-0.60	0.94	-3.72	-2.86	-2.45	-1.63

PF = punctal fluorescence, IPF = Inter-punctal fluorescence, FWHM = Full Width at Half Maximal, IPD = Inter-punctal Distance.
