# Supplementary material for: Profiling Synaptic Proteins Identifies Regulators of Insulin Secretion and Lifespan
Source: PLoS Genet. 2008 Nov 28;4(11):e1000283. doi: 10.1371/journal.pgen.1000283 (PMC2582949; doi:10.1371/journal.pgen.1000283)
Supplement: Text S1 — Supporting information. Calculating the importance of each parameter to a cluster. (0.16 MB PDF) [file pgen.1000283.s007.pdf]

Supporting Information

Calculating the importance of each parameter to a cluster.

To assess the importance of a parameter for the similarity between two genes, we asked how removal of that parameter affected the Pearson's Correlation R value between a pair of genes. First we computed the R value for the gene pair. Next we removed a single parameter p and re-computed the new R value, R'_p. The raw contribution of the parameter p to the similarity between the two genes would be reflected in how much the R value changed when that parameter was dropped, and would correspond to $\Delta R_p = R - R'_p$.

For each cluster, the ΔR_p values were computed for all pairwise comparisons between all genes within the cluster. For each parameter p, the mean $\overline{\Delta R_p}$ for the cluster was taken as the raw score for its contribution to grouping genes in that cluster.

We also calculated a mean background score, $\overline{\Delta R}_{p_{background}}$ for each cluster, corresponding to how much each parameter contributed to the similarity between genes within cluster to genes outside the cluster. This allowed us to focus on the distinctive phenotypes observed within a cluster by subtracting the background contribution for each parameter. Thus, parameters that contributed to unique similarity between genes in a cluster, rather than similarity among all genes were emphasized. The background score was obtained by calculating the means of the $\Delta R_{p_{background}}$ score obtained by computing the contribution of parameter p in the similarity between each gene within the cluster with each and every gene outside the cluster.

The score reflecting the contribution of parameter *p* to the similarity of genes within a given cluster would be given by $\overline{\Delta R}_p - \overline{\Delta R}_{p_{background}}$, with higher scores indicating greater importance of that parameter as a signature of the cluster. We call this the Clustering Contribution Score (CCS).

The parameters were ranked based on their CCS, with a rank of 1 being the most important. The cumulative CCS in rank order was computed, and the threshold rank for each cluster (i.e. the lowest ranking parameter that was still considered important to the cluster) was identified as the rank where the cumulative CCS exceeded 95% of the maximal cumulative CCS (Figure S1A).

For each cluster, we generated a subsets of the original dataset for clustering, consisting of only the top *n* ranked parameters (Figure S1A). In general, as *n* increased to the threshold rank, performance (as measured by the number clustering methods that identified the cluster) increased, and often exceeded the performance from analysis of the whole dataset. This suggested that by selecting only the most important parameters for each cluster, we were better able to identify genes that fit into that cluster, possibly by removing unrelated parameters that contribute to noise.

In the cluster consisting of *sad-1*, *syd-2* and *goa-1*, using only the parameters corresponding up to the threshold rank did not improve the clustering performance; additional parameters were required. The lower number of important parameters in this cluster (Figure S1A) suggested that this cluster is not as strong, likely due to less consensus in the important parameters obtained among the pairwise genes comparisons within this cluster. Analysis of the sub-cluster consisting of *sad-1* and *syd-2* (Figure S1B) indicate that clustering performance between these two genes were much more

robust, with more parameters contributing to their similarity. This analysis revealed that the membership of *goa-1* in this cluster is not as robust, and for this reason, we excluded it from the cluster with *sad-1* and *syd-2*.
